# Keratin 8 Is an Inflammation-Induced and Prognosis-Related Marker for Pancreatic Adenocarcinoma

**DOI:** 10.1155/2022/8159537

**Published:** 2022-07-27

**Authors:** Fei Xiong, Tong Guo, Xin Wang, Guanhua Wu, Wenzheng Liu, Qi Wang, Bing Wang, Yongjun Chen

**Affiliations:** ^1^Department of Biliary-Pancreatic Surgery, Tongji Hospital, Tongji Medical College, Huazhong University of Science and Technology, Wuhan, Hubei Province, China; ^2^Departement of Pediatric Surgery, Wuhan Children's Hospital, Tongji Medical College, Huazhong University of Science and Technology, Wuhan, Hubei Province, China

## Abstract

Pancreatic ductal adenocarcinoma (PDAC) is one of the highest-grade malignancies in the world. More effective biomarkers and treatment plans are necessary to improve the diagnosis rate and clinical outcome. The oncogenesis of PDAC is influenced by several factors, including chronic pancreatitis (CP). Keratin 8 (KRT8) is an important member of the keratin protein family and plays a role in regulating the cellular response to stress stimuli and mediating inflammatory reactions. However, the role of KRT8 in pancreatitis and PDAC is still poorly understood. Here we assessed the differentially expressed genes (DEGs) by bioinformatic methods with expression profiles available online for a caerulein-induced mouse model and human PDAC tissue. The prognostic value was evaluated by Kaplan–Meier analysis and Cox regression analysis. The diagnostic value was evaluated by Receiver Operating Characteristic analysis (ROC). The function of the genes was predicted by protein-protein interaction analysis, correlation analysis, and GO analysis. The conclusion was further validated in rat pancreatitis model, human tissue, and PDAC cell lines, including immunohistochemical staining (IHC), CCK-8 assay, wound healing assay, and flow cytometry. KRT8 was found to be upregulated in murine pancreatitis tissue, human CP tissue, and human PDAC tissue. High expression of KRT8 had a negative impact on the prognosis of PDAC patients. KRT8 was predicted to be involved in the regulation of the migration and viability of PDAC cells, which was validated in PDAC cell lines. Knockdown of KRT8 impaired the migration and proliferation and induced apoptosis in PDAC cell lines. In conclusion, keratin 8 is an inflammation-induced molecule and could serve as a diagnostic and prognostic marker for PDAC patients. More studies are needed for further validation from the perspective of precision and individualized medicine.

## 1. Introduction

Pancreatic ductal adenocarcinoma (PDAC) is one of the highest-grade malignancies in the world, ranking fourth in cancer-related deaths. The overall 5-year survival rate of PDAC is approximately 9% [1]. Although the incidence rate is relatively low, the mortality rate of PDAC is high due to its absence of early symptoms, high invasive ability, and delayed diagnosis [2, 3]. Surgical treatment is the best treatment for PDAC patients in the early stages. For those in the advanced stage, there are further treatment options such as chemotherapy, radiotherapy, immunotherapy, and targeted therapy. However, PDAC patients have poor prognosis due to their poor response and even resistance to these treatments [4]. Therefore, effective biomarkers and treatment plans are necessary for improving the diagnostic rate and clinical outcome.

The oncogenesis of PDAC is influenced by several factors, including chronic pancreatitis (CP). Patients with CP have a 13.3 times greater relative risk of developing PDAC than the general population [5, 6]. CP is characterized by recurrent episodes of pancreatic inflammation and persistent inflammatory injury, which can lead to pancreatic fibrosis (PF) and pancreatic dysfunction [5]. The risk factors for CP include smoking, alcohol consumption, and heritable mutations [7]. In addition, CP is highly correlated with a medical history of recurrent acute pancreatitis (AP). According to a meta-analysis, approximately 10% of the patients with a first episode of AP and 36% of those with recurrent AP ultimately developed CP [8]. Despite advances in the study of CP and AP, including clinical trials and animal experiments, little is known about the molecular mechanisms by which pancreatitis is involved in the development of PDAC.

Keratin 8 (KRT8) is an important member of the keratin protein family and forms a functional dimer with keratin 18 (KRT18) to maintain the structural integrity of epithelial cells [9]. In addition, KRT8 also plays a role in regulating the cellular response to stress stimuli. For example, KRT8 could mediate cisplatin-induced cytotoxicity and resistance to necrotic cell death [10, 11]. KRT8 is regarded as an epithelial marker in tumour pathology partly because it is highly expressed in epithelial structures [12, 13]. In the context of gastric cancer and prostate cancer, KRT8 was found to participate in the regulation of epithelial-mesenchymal transition (EMT) [14–16]. Additionally, KRT8 was reported to be a mediator of inflammatory reactions. KRT8 was found to participate in lung tissue regeneration and fibrosis after the inflammatory injury [17]. Gene polymorphism of KRT8 was found in some pancreatitis patients based on a large population-based study [18]. In murine pancreatitis models, KRT8 was significantly upregulated in pancreas tissue compared with the control group [19]. However, the role of KRT8 in pancreatitis and PDAC is still poorly understood.

Animal models are widely used for the study of pancreatitis, for example, caerulein-induced mouse models and sodium taurocholate- (STC-) induced rat models [20, 21]. In this study, we aimed to find a biomarker that met the following requirements. (1) The expression of the marker should be significantly upregulated or downregulated as the disease develops from pancreatitis to PDAC. (2) The marker should be used for the diagnosis of PDAC. (3) The marker should indicate the prognosis of PDAC patients. Here, we assessed differentially expressed genes (DEGs) by bioinformatic methods with the expression profiles available online for a caerulein-induced mouse model and human PDAC tissue based on the etiology of PDAC. Then, we focused on the role of KRT8 as a diagnostic and prognostic factor for PDAC patients. The expression of KRT8 was validated in another animal model, human tissue samples. The function of KRT8 was validated with PDAC cell lines. We found that KRT8 was upregulated in the context of pancreatitis and PDAC in an incremental manner and could serve as a diagnostic and prognostic factor for PDAC patients.

## 2. Materials and Methods

### 2.1. Microarray or Public Dataset Analysis

Three microarray datasets from murine AP models (GSE3644, GSE109227, and GSE121038) [22–24], one microarray dataset from murine CP models (GSE41418) [25], two microarray datasets from murine models with time-continuous observation (GSE40895 and GSE65146) [26, 27], and six microarray datasets from human PDAC and normal tissues (GSE15471, GSE16515, GSE32676, GSE62452, GSE71729, and GSE71989) [28–31] were downloaded from the Gene Expression Omnibus database (GEO; https://www.ncbi.nlm.nih.gov/geo) (detailed in Table S1). Datasets from The Cancer Genome Atlas (TCGA) and Genotype-Tissue Expression (GTEx) database were downloaded from the UCSC Xena database (https://xenabrowser.net/) [32].

Downloaded datasets were processed with the “limma” package in R 3.6.3 to identify the DEGs (https://www.r-project.org) [33]. False-positive results were avoided by adjusted *P* values (adj. *P*) by Benjamini–Hochberg analysis. The fold change value was calculated on a logarithmic scale (logFC). DEGs were defined with the cut-off values: adj. *P* < 0.05 and |*logFC*| > 1. Among the datasets, four datasets (GSE16515, GSE15471, GSE32676, and GSE71989) were processed by the same platform. The batch effect was corrected with the “sva” package [34]. The results were visualized by the “ggpubr” and “pheatmap” packages. An online Venn diagram tool was used to identify the intersections among different gene sets (http://bioinformatics.psb.ugent.be/webtools/Venn). Protein-protein interaction analysis was performed by the STRING database, and the minimum required interaction score was set as 0.4. The result was visualized by Cytoscape 3.7.2.

### 2.2. Gene Function Enrichment Analysis

Gene ontology (GO) analysis and Kyoto Encyclopedia of Genes and Genomes (KEGG) analysis were performed by the Database for Annotation, Visualization and Integrated Discovery (DAVID; https://david.ncifcrf.gov/tools.jsp) [35, 36]. The cut-off criterion to identify significantly enriched functions was defined as adj. *P* < 0.05. The results were visualized by the “GOplot” package [37]. The function of a single gene was retrieved from the PathCards database (https://pathcards.genecards.org) [38].

### 2.3. Cell Culture, siRNA Transfection, and Lentivirus Transduction

The human PDAC cell lines (SW-1990, Panc-1, MIA-PACA-2, and BxPC-3) and normal pancreas cell line HPDE were maintained in our laboratory. All the cell lines were cultured in DMEM supplemented with 10% foetal bovine serum, 100 U/mL penicillin, and 100 *μ*g/mL streptomycin at 37°C in a humidified incubator with 5% CO_2_. PDAC cells were seeded in six-well culture plates. After a 24 h culture, the complete medium was replaced. PDAC cells were transfected with silencing siRNA (siRNA-KRT8-1 and siRNA-KRT8-2) (50 nM) and nonsense siRNA (siRNA-KRT8-NS) (50 nM) with Lipofectamine 2000 (Thermo Fisher Scientific, USA). After 24 hours, the medium was replaced. After another 24 hours, the total RNA and total protein were then extracted. The siRNA used in the study was synthesized by Sangon Biotech, China. The sequences were as follows: siRNA-KRT8-1, sense, 5′-GCCUCCUUCAUAGACAAGGUATT-3′, antisense, 5′-UACCUUGUCUAUGAAGGAGGCTT-3′ and siRNA-KRT8-2, sense, 5′-GAGGACUUCAAGAACAAGUAUTT-3′, antisense, 5′-AUACUUGUUCUUGAAGUCCUCTT-3′.

The lentivirus vectors which carried the same silencing sequence as the siRNA (LV-KRT8-1 and LV-KRT8-2), and the corresponding negative control (LV-KRT8-NC) was constructed by Genechem, China. SW-1990 and Panc-1 cells were transfected with specific lentiviruses at a multiplicity of infection (MOI) of 20 with HistransG (Genechem) for 24 h. The cells were cultured in complete medium. After two days, the cells were treated with 1 *μ*g/ml of puromycin for establishment of stable cell lines.

### 2.4. Animal Models

This study was approved by the Animal Experimentation Ethics Committee of Tongji Medical College. Animal care and experimental procedures were performed according to the criteria outlined in current NIH guidelines (https://oir.nih.gov/sourcebook/ethical-conduct/research-ethics/nih-guidelines). Male Sprague-Dawley rats (280-320 g, with 3-4 weeks of age) and female BALB/C nude mice with 5 weeks of age were purchased from Charles River, China, and housed in a specific pathogen-free (SPF) environment.

The rat pancreatitis model was induced by 5% sodium taurocholate (1 mL/kg; Sigma, USA) according to the protocols provided previously with minor changes [39]. A total of 14 rats were evenly and randomly divided into two groups. After the rats were fasted for 6 hours, the models were established via surgery. The steps were as follows: (1) anesthetization with pentobarbital (3%), (2) celiotomy and dissection of the biliopancreatic duct and the hepatic duct, (3) clamping the biliopancreatic duct and the hepatic duct. The hepatic duct was closed by a small bulldog clamp. (4) Retrograde infusion into the biliopancreatic duct with 5% sodium taurocholate (1 mL/kg). The biliopancreatic duct was cannulated through the duodenum. (5) Removal of the clamp, sewing up the incision and anesthesia recovery. Rats in the control group underwent the same surgery and were treated with the same volume of saline solution. The treated rats were kept fasting and closely monitored after surgery. After fasting for 8 hours, the rats were sacrificed and the pancreas tissue was collected for further analysis.

Fifteen female BALB/C nude mice were evenly and randomly divided into three groups (LV-KRT8-NC, LV-KRT8-1, and LV-KRT8-2). Individual mice were injected subcutaneously with 2 × 10^6^ Panc-1 cells each group into the upper-right flank. All mice were sacrificed at 20 days postinoculation, and the subcutaneous tumours were excised and measured. The volume was calculated by this formula: volume = 0.5 × length × width^2^.

### 2.5. Tissue Samples, Histology, and Immunohistochemical Staining (IHC)

Twenty-two normal pancreas samples, 27 CP samples, and 33 PDAC samples were obtained from the PDAC patients at Tongji Hospital of Huazhong University of Science and Technology, China. All patients involved in this study were diagnosed with pancreatic masses. According to the result of the postoperative pathological analysis, three types of tissues were classified for further analysis based on tissue morphology and inflammatory infiltration. All the samples had definite pathological diagnosis by more than three pathologists in Tongji Hospital. This study was approved by the Ethics Committee of Tongji Hospital. The tissue samples were cleaned and fixed with 4% paraformaldehyde and subjected to dehydration, embedding, and sectioning at 5 *μ*m thickness. Then, the samples were stained with haematoxylin and eosin (HE).

IHC was performed by Elivision TMsuper HRP (Mouse/Rabbit) IHC Kit (KIT-9922; Biotechnologies, China). Before IHC staining, tissue samples were deparaffinized, rehydrated, and subjected to antigen retrieval. After blocking with 5% bovine serum albumin (BSA), tissue samples were incubated with primary antibodies overnight at 4°C. Tissue samples were incubated with horseradish peroxidase- (HRP-) labelled secondary antibodies the next day, followed by DAB staining. The intensity of staining (0: negative, 1: weak, 2: moderate, and 3: strong) and the percentage of positive cells (0: negative, 1: 1-25%, 2: 26%-50%, 3: 51-75%, and 4: 76%-100%) were scored in a blinded manner. The IHC score was calculated as the product of the intensity score and the percentage of positive cells. Anti-cytokeratin 8 antibody (ab53280; Abcam, UK) was used for IHC.

### 2.6. RNA Extraction and Quantitative Real-Time PCR (RT–qPCR)

Total RNA was extracted from fresh PDAC cell line samples and human tissues samples with RNA Isolater Total RNA Extraction Reagent (Vazyme, China) and reverse-transcribed into cDNA using HiScript III RT SuperMix for qPCR (+gDNA wiper) (Vazyme) according to the manufacturer's instructions. Then, quantitative PCR was performed using ChamQ Universal SYBR qPCR Master Mix (Vazyme) in an iQ5™ quantitative PCR detection system (Bio-Rad, USA). The primers used in the study were as follows: KRT8, 5′-CAGAAGTCCTACAAGGTGTCCA-3′ and 5′-CTCTGGTTGACCGTAACTGCG-3′, KRT18, 5′-TCGCAAATACTGTGGACAATGC-3′ and 5′-GCAGTCGTGTGATATTGGTGT-3′, YWHAZ, 5′-TGATCCCCAATGCTTCACAAG-3′ and 5′-GCCAAGTAACGGTAGTAATCTCC-3′, LAD1, 5′-GATACCACACGGCCATACGG-3′ and 5′-GAGCCACGAATAACTCAGTGC-3′, and GAPDH, 5′-GGAGCGAGATCCCTCCAAAAT-3′ and 5′-GGCTGTTGTCATACTTCTCATGG-3′. The data was analysed using the 2^−∆∆Ct^ method.

### 2.7. Western Blot

Fresh PDAC cell samples were lysed in RIPA buffer containing protease inhibitor cocktail (Roche, Switzerland). The concentrations of the cell samples were determined with the bicinchoninic acid (BCA) method after the centrifugation of the cell lysates [9]. Individual protein samples (30 *μ*g per lane) were separated by sodium dodecyl sulfate-polyacrylamide gel electrophoresis (SDS-PAGE) and then transferred to the polyvinylidene difluoride membranes (PVDF; Millipore, USA). The membranes were incubated with primary antibodies overnight at 4°C after blocking in 5% skimmed dry milk in TBST. Then, the membranes were incubated with HRP-conjugated anti-rabbit and anti-mouse IgG (BOSTER, China), and the results were visualized by ECL (32106; Thermo Fisher Scientific). Anti-E-cadherin antibody (20874-1-AP), anti-N-cadherin antibody (22018-1-AP), anti-vimentin antibody (10366-1-AP), anti-caspase 3 antibody (19677-1-AP), and anti-BCL-2 antibody (12789-1-AP) were bought from Proteintech, USA. Anti-cleaved caspase 3 antibody (ab32042) and anti-cytokeratin 8 antibody (ab53280) were bought from Abcam. Anti-GAPDH antibody (BM1623) and anti-BAX antibody (BA0315-1) were bought from BOSTER.

### 2.8. CCK-8 Assay

PDAC cells of different groups (siRNA-KRT8-NS, siRNA-KRT8-1, and siRNA-KRT8-2 groups) were plated in 96-well plates (1.5 × 10^3^ per well). The proliferation ability was evaluated after 0, 24, 48, and 72 hours. Ten microliters of CCK-8 solution from a Cell Counting Kit (40203ES60; Yeasen, China) was added to each well with 90 *μ*L of DMEM, followed by incubation for two hours. The absorbance at 450 nm was measured with a MULTISKAN FC microplate reader (Thermo Fisher Scientific).

### 2.9. Flow Cytometry

The apoptosis of PDAC cells was examined using an Annexin V-FITC/PI apoptosis kit (70-AP101-100; MultiSciences, China). PDAC cells were collected (2 × 10^5^ per well) and stained with Annexin V-FITC/PI. The positive control group was treated by heat shock (55°C, 10 min) and stained with Annexin V-FITC/PI. Then, the percentage of apoptotic cells was evaluated by flow cytometry in a Becton-Dickinson FACScan System (Franklin Lakes, USA). Cell cycle was analysed by Cell cycle staining Kit (CCS012; MultiSciences). PDAC cells were collected (2 × 10^6^ per well) and stained with PI. Then, the cell cycle distribution was evaluated by flow cytometry.

### 2.10. Wound Healing Assay

PDAC cells from different groups (siRNA-KRT8-NS, siRNA-KRT8-1, and siRNA-KRT8-2 groups) were plated in 6-well plates and cultured until almost 100% confluency. A pipette tip of 20 *μ*L was used to generate a linear scratch. Next, the complete medium was removed. The treated cells were cultured in serum-free DMEM. Five time points (0 h, 12 h, 24 h, 36 h, and 48 h) were used for the observation of the migration of the cells. The result was photographed under an inverted microscope. The cellular migration rate was used to evaluate the migration ability of PDAC cells (*cellular* *migration* *rate* = (*scratched* *area* (0 *h*) − *scratched* *area* (*time* *points*)/*scratched* *area* (0 *h*)).

### 2.11. Statistical Analyses

The data are presented as the means ± standard error of the mean (SEM). The data are representative images from three separate experiments. The differences between two groups were examined by the Student *t*-test. The differences among three or more groups were examined by ANOVA. The survival and phenotype data of TCGA samples were obtained from the UCSC Xena database. The survival of PDAC patients was evaluated by Kaplan–Meier curves. The patients were divided into a high expression group and a low expression group according to the median expression level of KRT8. The cut-off criterion used to determine statistical significance was log-rank *P* < 0.05. The results were visualized by KM plotter (http://kmplot.com/analysis) [40]. Univariate and multivariate Cox regression analyses were performed by the “survival” package. The chi-square test was used to analyse the correlation between KRT8 expression and clinicopathological features (*P* < 0.05). Pearson correlation coefficient (Pearson *r*) was used to assess the bivariate correlations between the expression of different genes (*P* < 0.05). The result was visualized by the “corrplot” package. Receiver Operating Characteristic analysis (ROC) was performed by the “pROC” package [41]. The diagnostic value was evaluated by area under curve (AUC). All statistical analyses were performed by GraphPad Prism 8.0 (GraphPad, USA) and R 3.6.3.

## 3. Results and Discussion

### 3.1. Identification of AP-Related DEGs in Murine Models

The workflow is shown in Figure S1. In the prediction part, the DEGs from the murine expression profiles were filtered by the human PDAC microarrays, prognostic analysis, and function prediction. Then, the conclusion was validated in murine pancreatitis model, human tissues, and PDAC cell lines in validation part (Figure S1). As pancreatitis is one of the etiological factors of PDAC, we first analysed the microarrays of pancreatitis samples. Due to the lack of human pancreatitis microarrays, we chose the microarrays of murine animal models for further analysis. Three microarray datasets were used for the identification of DEGs in AP tissue compared with normal pancreas tissue. A total of 327 upregulated DEGs and 141 downregulated DEGs were found in GSE3644 (Figure S2A). A total of 1333 upregulated DEGs and 305 downregulated DEGs were found in GSE109227 (Figure S2B). A total of 786 upregulated DEGs and 656 downregulated DEGs were found in GSE121038 (Figure S2C). The intersection of upregulated DEGs and downregulated DEGs was calculated among the three datasets. A total of 129 common upregulated DEGs and 19 common downregulated DEGs were found (Figure S2D).

Gene function enrichment analysis was performed with the common DEGs. In GO analysis, the results of the cellular component (CC) analysis indicated that 16 DEGs showed enrichment of the term “cell-cell adherens junction” (GO:0005913) (Table S2). Similarly, the related term “cadherin binding involved in cell-cell adhesion” (GO: 0098641) was found enriched in the molecular function (MF) analysis (Figure S2E). “Tight junction” (mmu04530) and “adherens junction” (mmu04520) were found enriched in the KEGG analysis (Figure S2F, Table S3), indicating that AP might influence the ratio of surface molecules and the adhesion ability of pancreas cells.

### 3.2. Identification of CP-Related DEGs in Murine Models

GSE41418, containing the sequencing data from two substrains of mice, was used for the identification of CP-related DEGs. A total of 1685 upregulated DEGs and 150 downregulated DEGs were found in the Jackson mice data (Figure S3A). A total of 1974 upregulated DEGs and 188 downregulated DEGs were found in the Harlan mice data (Figure S3B). We found 1109 common upregulated DEGs and 86 common downregulated DEGs by determining the overlapping genes (Figure S3C).

The common DEGs were then analysed by GO and KEGG analyses. Some GO terms related to surface molecules were found enriched, such as “cell surface” (GO:0009986) and “cell-cell adherens junction” (GO:0005913) in the CC analysis (Table S4). Intriguingly, some terms related to cell proliferation were identified, such as “cell cycle” (GO:0007049, mmu04110) and “DNA replication” (GO:0006260, mmu03030) in the GO and KEGG analyses (Figure S3D, S3E, Table S5), indicating that unlike AP, CP might significantly influence not only the adhesion but also the viability of pancreas cells.

### 3.3. Identification of DEGs from Continuous Observation Data

**Figure 1:**
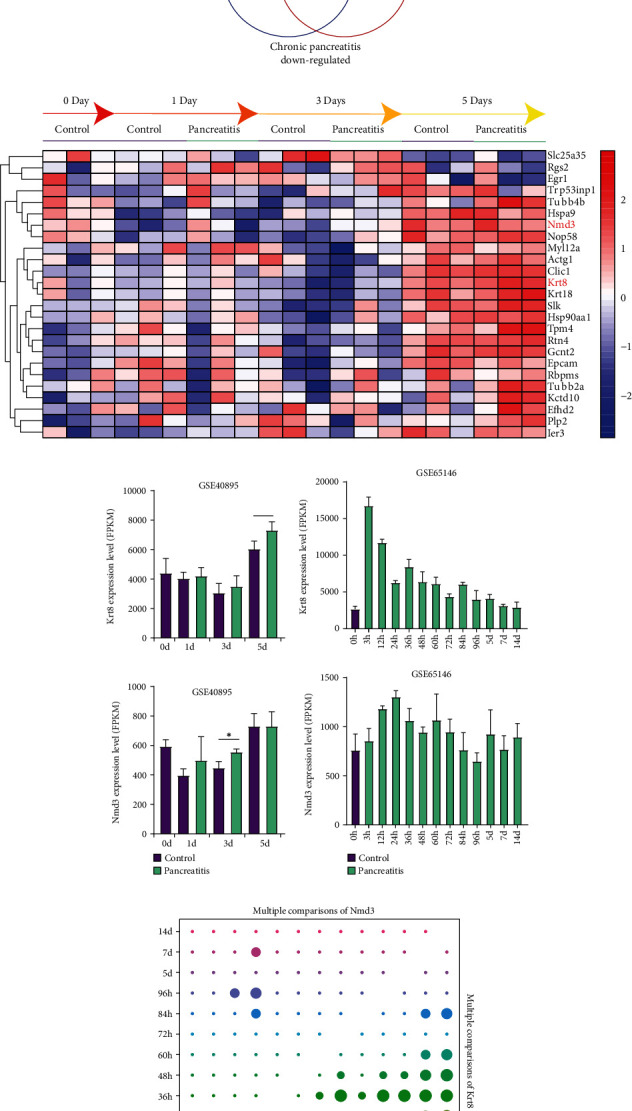
Presentation of the DEGs in continuous observation microarrays. (a) Venn diagram for the intersection of the DEGs between AP tissue and CP tissue. (b) The heatmap for the presentation of common DEGs in GSE40895. (c) The bar chart for the expression of Krt8 and Nmd3 in GSE40895. (d) The bar chart for the expression of Krt8 and Nmd3 in GSE65146. (e) The matrix diagram for the result of multiple comparisons of Krt8 and Nmd3 in GSE65146 by ANOVA. Larger bubbles represent smaller *P* values. ^∗^*P* < 0.05, ^∗∗^*P* < 0.01, ^∗∗∗^*P* < 0.001, and ^∗∗∗∗^*P* < 0.0001.

**Figure 2:**
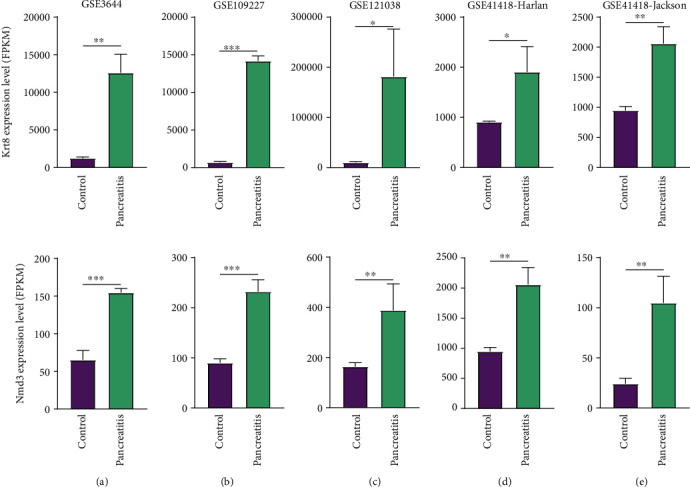
Presentation of the expression of Krt8 and Nmd3. (a) GSE3644. (b) GSE109227. (c) GSE121038. (d) GSE41418 Harlan group. (e) GSE41418 Jackson group. ^∗^*P* < 0.05, ^∗∗^*P* < 0.01, and ^∗∗∗^*P* < 0.001.

Two large datasets, GSE40895 and GSE65146, both of which contain the results of continuous observation of murine models after caerulein injection, were included to further refine the DEGs. First, we calculated the intersection between the AP and CP DEGs and found 24 common upregulated DEGs and 1 common downregulated DEG (Figure 1(a)). These genes might be involved in the long-term inflammation damage to pancreas tissue. Then, these 25 DEGs were validated with GSE40895 (Figure S4). The expression of these DEGs is shown in Figure 1(b). We found that only the expression of murine Krt8 and Nmd3 (NMD3 ribosome export adaptor) was significantly changed upon the occurrence of pancreatitis (Krt8 on day 5, *P* = 0.048; Nmd3 on day 3, *P* = 0.016) (Figure 1(c)). Subsequently, we analysed the GSE65146 dataset (Figure 1(d)). The expression of Krt8 and Nmd3 differed significantly from that in the control samples, though the difference in Nmd3 expression could only be observed in a small number of samples (Figure 1(e)). The expression of these two genes in the microarrays mentioned above (GSE3644, GSE109227, GSE121038, and GSE41418) is shown in Figure 2. Interestingly, we found that caerulein-induced upregulation of Krt8 peaked after 3 hours and decreased with time afterwards (Figures 1(d) and 1(e)), which is similar to a famous biomarker of pancreatitis, amylase [42]. In contrast, the upregulation of Nmd3 seemed to be irregular.

### 3.4. Comparison of KRT8 Expression in Human PDAC Microarray Datasets

**Figure 3:**
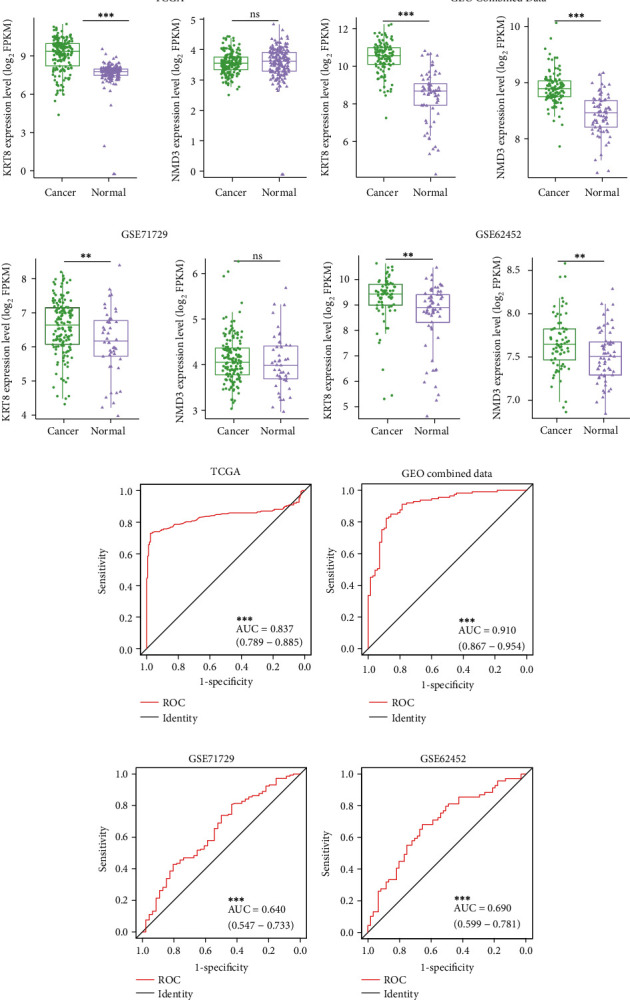
Presentation of KRT8 expression in human PDAC tissue and normal pancreas tissue. (a) TCGA. (b) GEO combined data. (c) GSE71729. (d) GSE62452. ROC curves were used for the evaluation of the diagnostic value. (e) TCGA. (f) GEO combined data. (g) GSE71729. (h) GSE62452. ^∗∗^*P* < 0.01. ^∗∗∗^*P* < 0.001.

Human KRT8 and NMD3 were similar to murine Krt8 and Nmd3, respectively. As CP is an important cause of PDAC, we determined to compare the difference in KRT8 expression between PDAC tissue and normal tissue. We analysed the mRNA sequencing data obtained from TCGA and GTEx (178 PDAC samples and 171 normal pancreas samples) and found that the expression of KRT8 was higher in cancerous tissue (7.640 ± 0.080 versus 9.067 ± 0.093, *P* < 0.001) (Figure 3(a)). To provide more evidence, we retrieved related microarray datasets from GEO. Four GEO microarray datasets were combined (113 PDAC samples and 70 normal pancreas samples). The microarrays that treated with different platforms, such as GSE71729 and GSE62452, were also analysed. We found that the expression of KRT8 in human PDAC tissue was significantly higher than that in normal pancreas tissue (GEO combined data, 8.529 ± 0.158 versus 10.480 ± 0.077, *P* < 0.001; GSE71729, 6.205 ± 0.139 versus 6.636 ± 0.065, *P* < 0.01; and GSE62452, 8.638 ± 0.164 versus 9.288 ± 0.116) (Figures 3(b)–3(d)). The expression of NMD3 was also examined, but significant differences were observed only in two out of four microarrays.

The value of KRT8 in the differentiation between PDAC and normal cases was evaluated by ROC curves. The AUC of TCGA dataset is 0.837 (0.789-0.885) (Figure 3(e)). The AUC of GEO combined data is 0.910 (0.867-0.954) (Figure 3(f)). The diagnostic value of KRT8 was high in these two datasets (AUC: 0.8-1.0). The AUC of GSE71729 and GSE62452 was 0.640 (0.547-0.733) and 0.690 (0.599-0.781) (Figures 3(g) and 3(h)). The diagnostic value of KRT8 was relatively low in these two datasets (AUC: 0.6-0.7).

### 3.5. Evaluation of the Correlation between KRT8 Expression and the Prognosis of PDAC Patients

**Figure 4:**
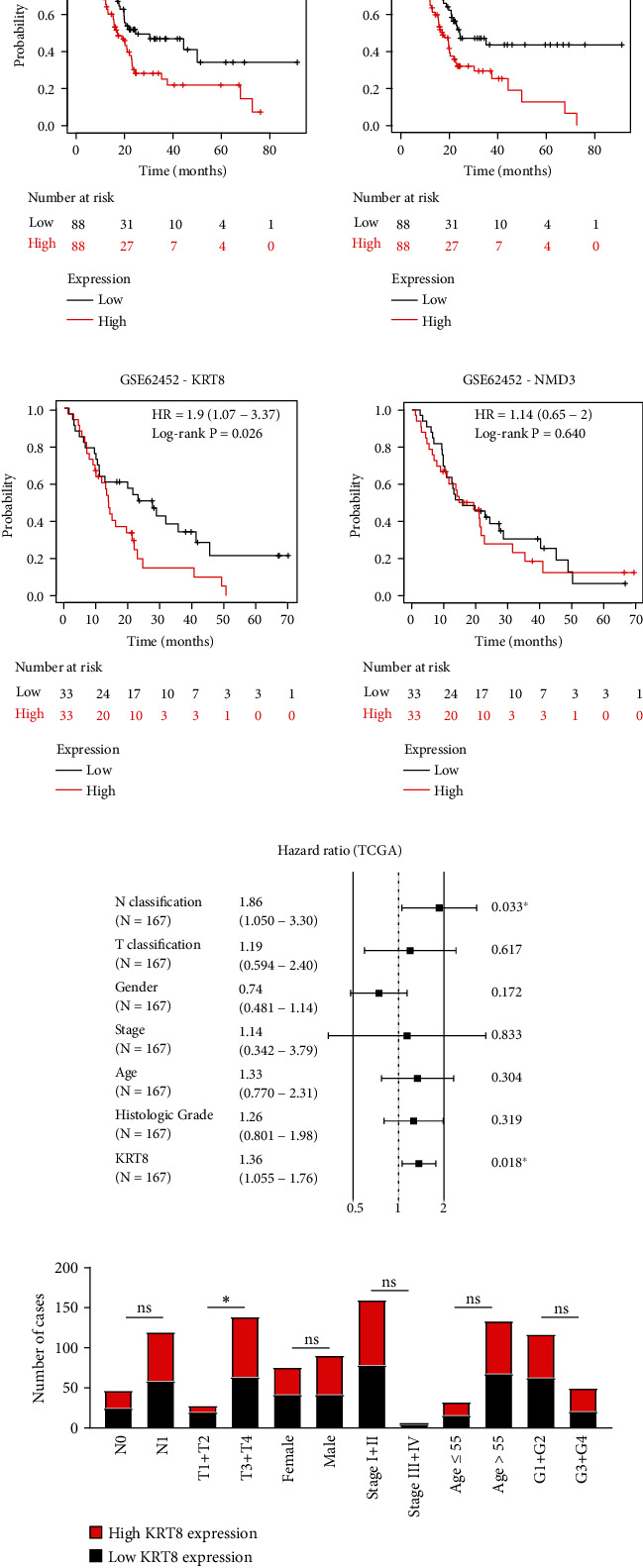
Association of KRT8 expression and the prognosis of PDAC patients. (a) Kaplan–Meier curves and log-rank test results for PDAC patients in TCGA. (b) Kaplan–Meier curves and log-rank test results for PDAC patients in GSE62452. (c) The result of multivariate Cox regression analyses. (d) The bar chart for the result of the chi-square test for the correlation between KRT8 expression and clinicopathological features. ^∗^*P* < 0.05.

To investigate the potential role of KRT8 as a prognostic factor, we performed survival analysis with the data from TCGA and GSE62452. The results revealed that higher expression of KRT8 had a negative impact on the prognosis of PDAC patients (Figures 4(a) and 4(b)). However, we failed to find any correlation between NMD3 expression and the overall survival of PDAC patients with the data in GSE62452 (Figures 4(a) and 4(b)). In summary, after comprehensive consideration, KRT8 might be a better biomarker than NMD3.

Next, we performed univariate Cox regression analyses with 176 patients from TCGA. The results showed that N classification (*P* = 0.004) and KRT8 expression (*P* = 0.006) were significant risk factors for PDAC patients (Table 1). The results of multivariate Cox regression analyses revealed that N classification (*P* = 0.033) and KRT8 expression (*P* = 0.018) were independent predictors of PDAC survival (Figure 4(c) and Table 1). Then, we analysed the correlation between KRT8 expression and the clinicopathological features of PDAC. The results showed that KRT8 expression was correlated with T classification (*P* = 0.014) (Figure 4(d) and Table 2).

### 3.6. Prediction of KRT8 Functions in PDAC

**Figure 5:**
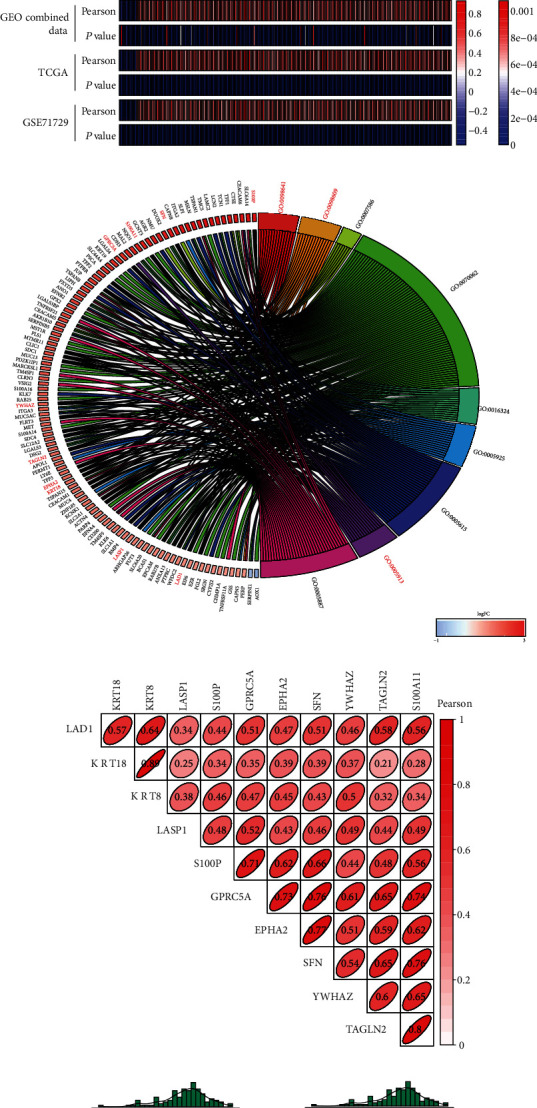
Prediction of KRT8 function in the context of PDAC. (a) Venn diagram for the KRT8-correlated genes in three microarrays. (b) The heatmap for the common KRT8-correlated genes in the intersection. (c) The circle plot of the result of GO analysis of the common KRT8-correlated genes. The red words represent the intersection among different terms. (d) The correlation heatmap that presents the Pearson *r* for each bivariate correlation analysis. (e) Correlation figures of the genes with the top three Pearson *r* from high to low. ^∗∗∗^*P* < 0.001.

To study the potential function of KRT8 in PDAC, we selected the microarray datasets that contained more than 100 PDAC samples for further analysis (TCGA and GEO combined data and GSE71729). The Pearson *r* values between the expression of KRT8 and each DEG were calculated, and the genes that met the criteria were included (|*Pearson* *r*| > 0.3 and *P* < 0.05). We found 202 common genes that positively correlated with KRT8 and 11 common genes that negatively correlated with KRT8 (Figures 5(a) and 5(b)). The results of the GO analysis showed that three EMT-related terms, “cadherin binding involved in cell-cell adhesion” (GO:0098641; 14 genes), “cell-cell adherens junction” (GO:0005913; 15 genes), and “cell-cell adhesion” (GO:0098609; 14 genes), were enriched (Figure 5(c), Table S6). The common genes enriched for the three terms were identified, and 10 genes were preserved (Figures 5(c) and 5(d)). The genes with the top three Pearson *r* values from high to low were KRT18, LAD1, and YWHAZ (Figure 5(e)).

Then, we performed the protein-protein interaction analysis. The potential interaction with KRT8 was retrieved in STRING database, and the interaction network was constructed (Figure S5). The result showed that among these 213 genes, 16 genes were found to potentially interact with KRT8, including other members of the keratin family (KRT18, KRT19, and KRT80) and important membrane proteins (EPCAM, CDH3, CEACAM5, and DSG2). Therefore, these results suggested that KRT8 might be involved in the regulation of the membrane protein function which was often found to be disordered in the tumour lesions.

As mentioned above, KRT8 expression can be induced in inflammatory lesions. To explore the role of KRT8 in inflammation process and cell viability, we identified the function terms of 213 DEGs. We selected five inflammation-related terms (innate immune system, interferon, interleukin, B-cell receptor signalling pathway, and NF-*κ*B signalling pathway) and three cell viability-related terms (Akt signalling pathway, MAPK signalling pathway, and apoptosis). Twenty-nine genes were involved in innate immune system function. Additionally, significant genes involved in interferon pathway (e.g., IFI27, OAS1, and OASL), interleukin pathway (e.g., IL10RA, LCN2, and TFF3), B-cell receptor pathway (e.g., ACTN4, EZR, and PTPRC), and NF-*κ*B signalling pathway (e.g., CDCP1, CEACAM5, and LGALS3) were found to be correlated with KRT8 (Figure S1, Table S7). These pathways were often involved in the onset of pancreatitis. Some terms about cell viability were also figured out, such as Akt pathway (e.g., BMP4, CCND1, and EPHA2), MAPK pathway (e.g., CDH17, CDH3, and LAMB3), and apoptosis pathways (e.g., CAPN5, CAPN8, and DSG2) (Figure S6, Table S7). The genes with the top three relevance scores provided by the PathCards database are shown from high to low in Figure S7 and Figure S8. Therefore, as a potential inflammation-induced factor, KRT8 might influence the migration, proliferation, and apoptosis of PDAC cells.

### 3.7. Validation of KRT8 Expression in Rat Models and Human PDAC Tissue

**Figure 6:**
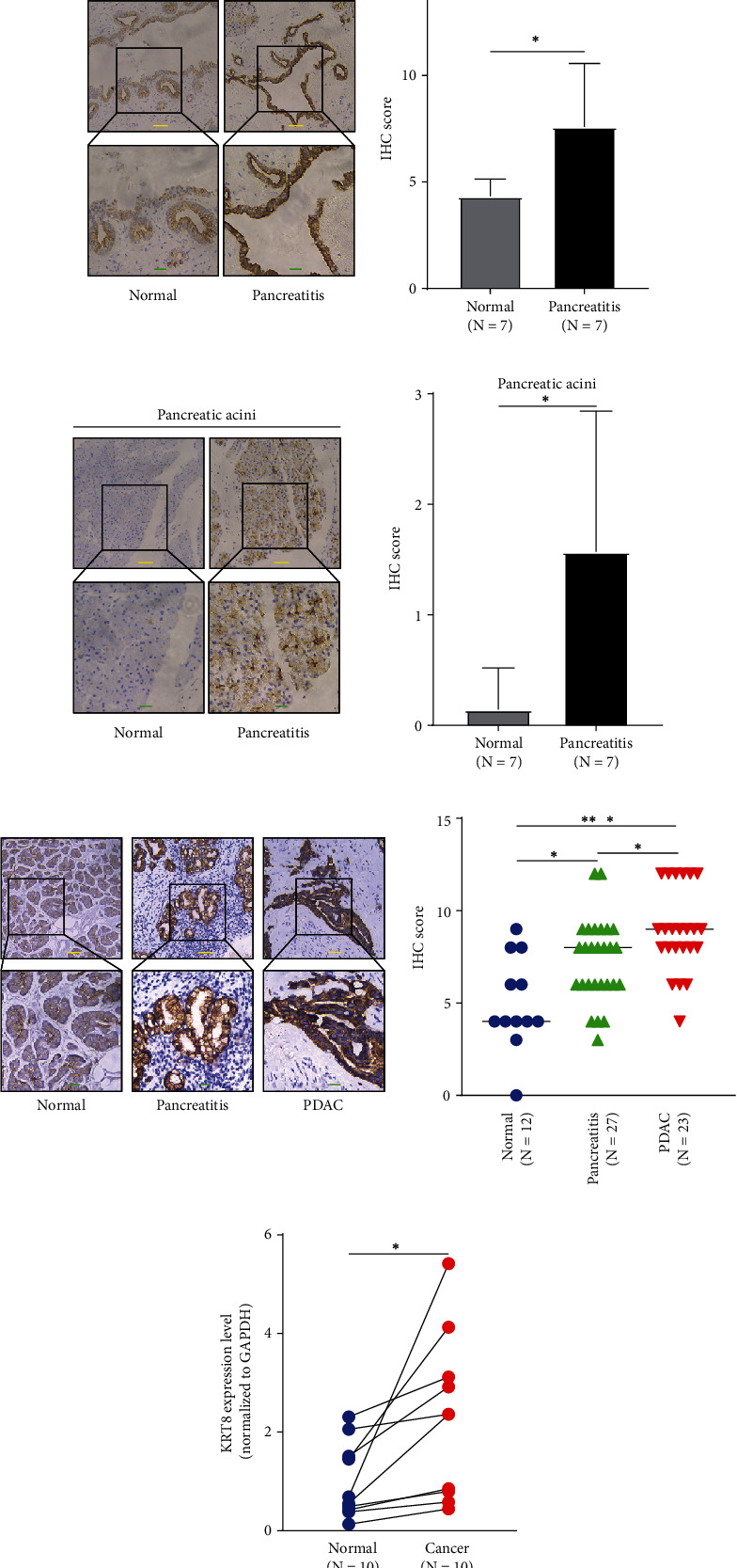
IHC and HE staining of rat models and human tissue samples. (a) The scheme of establishing the STC-induced rat pancreatitis models. (b) Some of the basic pathological changes of pancreas tissue in rat pancreatitis models. The yellow, blue, green, and red arrows represent the normal pancreatic ducts, blood vessels, pancreatic acini, and Langerhans' islets. (c) Presentation of the IHC staining of pancreatic ducts in pancreatitis tissue and control tissue. (d) Presentation of the IHC staining of pancreatic acinus in pancreatitis tissue and control tissue. (e) Presentation of the IHC staining of human normal pancreas tissue, pancreatitis tissue, and PDAC tissue. The IHC score was used to assess the expression of KRT8. (f) The RT-qPCR result of the paired tissues. (g) The Western blot result of the paired tissues. Blue bar, 100 *μ*m. Yellow bar, 50 *μ*m. Green bar, 20 *μ*m. ^∗∗∗^*P* < 0.001. ^∗^*P* < 0.05.

We performed IHC to validate the expression pattern of KRT8 in pancreatitis and PDAC tissues. We failed to obtain human AP tissue because mild AP is not an appropriate indication for surgery. Therefore, another murine model was selected for further analysis (Figure 6(a)). Typical pathological changes were observed by microscopy, e.g., local haemorrhage, local necrosis, residual pancreas, and inflammatory cell infiltration after the injection of STC (Figure 6(b)). Surprisingly, after IHC staining, we found that unlike the staining of human tissue, the staining of rat pancreatic ducts was obvious while the expression of KRT8 was hardly detected in rat pancreatic acini. Therefore, these two structures were analysed separately. We found that KRT8 expression in ducts and acini was both elevated upon STC treatment compared with that in the control group (ducts, *P* = 0.028 and acini, *P* = 0.015) (Figures 6(c) and 6(d)). A similar conclusion was reached with human tissue. KRT8 expression in CP tissue and PDAC tissue was higher than that in normal pancreas tissue (CP tissue versus normal tissue, *P* = 0.015 and PDAC tissue versus normal tissue, *P* < 0.001) (Figure 6(e)). Further analysis showed that the expression of KRT8 in PDAC was slightly higher than that in CP tissue (*P* = 0.042) (Figure 6(e)). Therefore, KRT8 was upregulated in pancreatitis and PDAC in an incremental manner. To provide more evidence, we performed Western blot and RT-qPCR with paired normal pancreas samples and PDAC samples. We found KRT8 was upregulated in PDAC tissues compared with normal pancreas tissues, in both mRNA and protein levels (Figures 6(f) and 6(g)). The information of the patients mentioned in this study was summarized in Table S8.

### 3.8. Validation of KRT8 Function in PDAC Cell Lines

**Figure 7:**
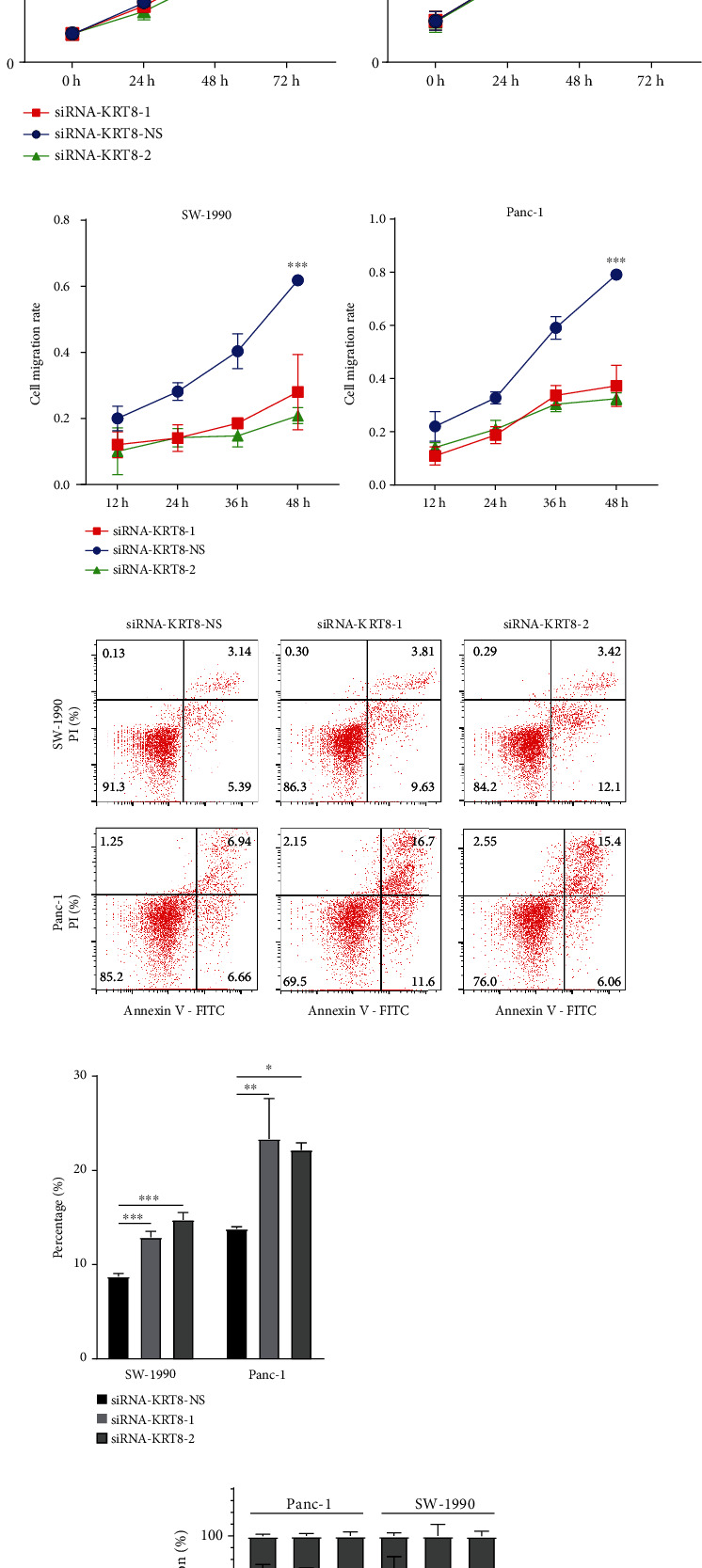
Examination of the migration and viability of PDAC cells upon KRT8 knockdown. (a) The difference in KRT8 expression among PDAC cells and normal pancreas cells. (b, c) Validation of the efficiency of KRT8 knockdown by RT-qPCR and Western blot. (c) Illustration of the result of CCK-8 assay. (d) Illustration of the result of wound healing assay. Cell migration rate was used to evaluate the migration ability of PDAC cells. (f) The result of flow cytometry and the difference of apoptotic cells. (g) The distribution of cell cycle status of PDAC cells. (h) The growth of Panc-1 tumours in nude mice (*N* = 5 per group). Red bar, 1 cm. ^∗∗∗^*P* < 0.001. ^∗∗^*P* < 0.01. ^∗^*P* < 0.05.

To validate the role of KRT8 in PDAC, we compared the expression of KRT8 among different PDAC cell lines. We found that compared with the normal pancreas cell line HPDE, the expression of KRT8 was found to be upregulated in three PDAC cell lines (Panc-1, SW-1990, and MIA-PACA-2) (Figure 7(a)). Panc-1 and SW-1990 were accepted for further analysis because the KRT8 expression of both cell lines was higher than that of MIA-PACA-2 (Figure 7(a)). We transfected SW-1990 and Panc-1 with nonsense siRNA and two distinct siRNAs. The expression level of KRT8 decreased significantly at the mRNA and protein levels (Figures 7(b) and 7(c)). CCK-8 assay revealed that the proliferation of PDAC cells was impaired upon KRT8 knockdown (Panc-1, *P* < 0.001 and SW-1990, *P* < 0.001) (Figure 7(d)). The migration of PDAC cells was inhibited upon KRT8 downregulation according to the results of the wound healing assay (Panc-1 after 48 h, 0.791 ± 0.007 versus 0.373 ± 0.045 versus 0.324 ± 0.013, *P* < 0.001 and SW-1990 after 48 h, 0.619 ± 0.006 versus 0.280 ± 0.066 versus 0.209 ± 0.014, *P* < 0.001) (Figure 7(e), Figure S9). Then, we performed flow cytometry and found that the ratio of apoptotic cells increased upon KRT8 knockdown (Panc-1, 13.79% ± 0.135% versus 23.360% ± 2.474% versus 22.17% ± 0.435%, *P* < 0.01 and SW-1990, 8.740% ± 0.195% versus 12.910% ± 0.353% versus 14.750% ± 0.445%, *P* < 0.001) (Figure 7(f), Figure S10). Cell cycle analysis was also performed, and we found KRT8 knockdown induced cell cycling arrest at G2/M phase in both cell lines (Panc-1, 14.280% ± 0.480% versus 21.320% ± 0.622% versus 24.190% ± 0.995%, *P* < 0.001 and SW-1990, 18.560% ± 0.772% versus 26.980% ± 2.875% versus 36.26 ± 1.205%, *P* < 0.01) (Figure 7(g)). The conclusion was further validated with nude mouse models, and we found KRT8 knockdown significantly inhibited the growth of implanted Panc-1 tumours (30.780 ± 7.588 mm^3^ versus 9.986 ± 0.978 mm^3^ versus 10.830 ± 2.821 mm^3^, *P* = 0.014) (Figure 7(h)).

**Figure 8:**
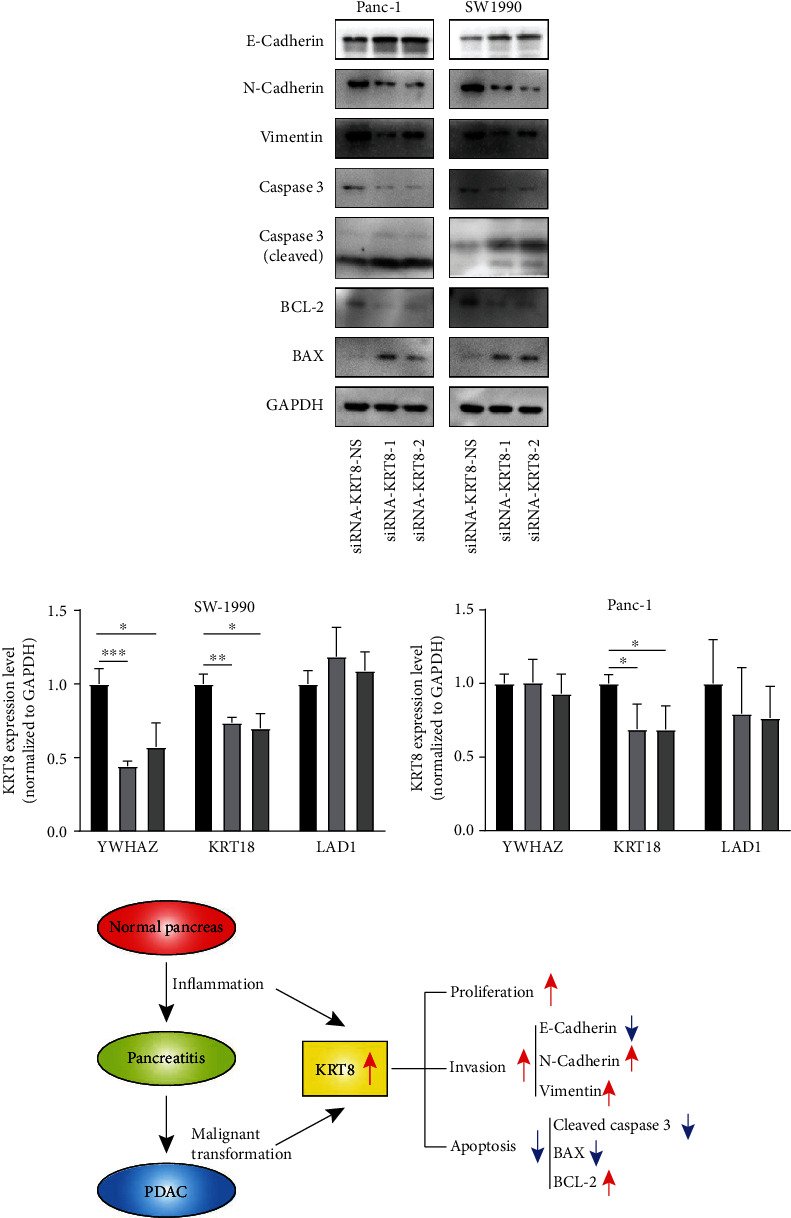
Validation of the function of KRT8. (a) Evaluation of the expression of EMT markers (E-cadherin, N-cadherin, and vimentin) and apoptosis mediators (caspase 3, BAX, and BCL-2) upon KRT8 knockdown. (b) The RT-qPCR result of three genes that correlated with KRT8 (KRT18, YWHAZ, and LAD1). (c) The schematic figure to illustrate the connection among KRT8 expression, inflammation, and tumour progression. KRT8 is upregulated in pancreatitis and PDAC in an incremental manner and influences the migration, proliferation, and apoptosis of PDAC cells. ^∗∗∗^*P* < 0.001. ^∗∗^*P* < 0.01. ^∗^*P* < 0.05.

To further elucidate the effect of KRT8 on the migration and viability of PDAC cells, we performed Western blot analysis. We found E-cadherin was upregulated upon the downregulation of KRT8 while N-cadherin and vimentin were both downregulated, indicating that the migration ability was impaired upon KRT8 knockdown (Figure 8(a)). In addition, the upregulation of BAX and cleaved caspase 3, the mediators of apoptosis, and downregulation of BCL-2, an antiapoptotic factor, were observed, indicating that apoptosis was induced by KRT8 knockdown (Figure 8(a)). To provide more evidence, we validated the result of the correlation analysis (Figures 5(d) and 5(e)). We found that only KRT18 was slightly downregulated upon KRT8 knockdown in both cell lines. YWHAZ downregulation was only found in SW-1990 cells (Figure 8(b)). As KRT18 could function by binding to KRT8, we performed survival analysis and found higher expression of KRT18 was related with worse prognosis of PDAC patients only in TCGA cohorts (Figure S11). In summary, KRT8 is an important regulator of the migration and viability of PDAC cells (Figure 8(c)).

## 4. Discussion

In this study, we applied a bioinformatics method to identify appropriate diagnostic and prognostic biomarkers for pancreatitis and PDAC. We first analysed the mRNA sequencing data of murine AP and CP models. Only two DEGs (KRT8 and NMD3) were identified. However, NMD3 was excluded from further analysis due to its weakness in expression pattern and prognostic value compared with KRT8. KRT8 was proven to be upregulated in PDAC tissue. The function of KRT8 in PDAC was predicted by protein-protein interaction analysis, correlation analysis, and GO analysis. We found that KRT8 might be involved in the regulation of migration and cell viability. The conclusions mentioned above were validated in rat models, human tissue, and PDAC cell lines (Figure S1).

Murine models have been widely used in studies of immunity, inflammation, and oncogenesis. Recently, the pathogenesis and therapeutic schedule of pancreatitis have been studied with murine pancreatitis models, such as the caerulein-induced mouse model and STC-induced rat model [20, 21]. In this study, six microarray datasets from the caerulein-induced mouse model were used, overcoming the problem of a lack of human AP and CP data. Pancreatitis could influence the transcriptome profiles of pancreas tissue and lead to different degrees of inflammation damage [22–25]. In this study, these datasets were reviewed and we found a great number of genes that were differently expressed between pancreatitis samples and control samples, including murine Krt8 and Nmd3 (148 genes in AP models and 1195 genes in CP models) (Figure S2A-D, S3A-C). The results of differential expression analysis and enrichment analysis revealed that both AP and CP could influence the function of membrane molecules and the adhesion ability of pancreatic cells, which might result from the response to oxidative stress and interaction with inflammatory mediators (Figure S2E, S2F, S3D, S3E) [22, 23]. Notably, CP was found to be involved in cell proliferation, which was not clearly found in AP (Figure S3D, S3E). This might explain why prolonged pancreatitis can gradually contribute to the development of PDAC. In this stage, the specific molecular mechanisms for limiting abnormal proliferation can be utilized as effective targets for preventing the malignant transformation, which remains to be determined.

Twenty-five DEGs were then picked out because of their overlapping roles in AP and CP (Figure 1(a)). The range of DEGs was further narrowed by analysis of continuous observation data. Only KRT8 and NMD3 were further analysed as both of them were significantly upregulated upon caerulein treatment, though the time points were different (Figures 1(b)–1(d) and 2(a)–2(d)). KRT8 was proven to have better potential as a biomarker of PDAC and pancreatitis for the following reasons. First, the upregulation of KRT8 in inflamed pancreas tissue appeared to be irritable upon caerulein injection, rather than a disorderly change (Figures 1(d) and 1(e)). Additionally, we noticed that KRT8 gradually reduces after a brief peak, which could be explained by the use of nonlethal dose of caerulein. After 3 hours, the proinflammatory effects of caerulein might be compensated by other molecular mechanisms. However, the level of KRT8 did not decrease to the baseline, indicating that the lesion could last for a long time (Figure 1(d)). Second, the differences in KRT8 expression between PDAC and normal pancreas tissue were statistically significant (Figures 3(a)–3(d)). Recent studies revealed that the overexpression of KRT8 could be also found in glioblastoma [43]. Interestingly, Nordgård et al. found that KRT8 was upregulated in the bone marrow aspirates from advanced PDAC patients, possibly caused by infiltrated PDAC cells, indicating the potential role of KRT8 in the formation of tumour microenvironment [44]. Consistent with this finding, we found the diagnostic value of KRT8 in differentiating normal cases from PDAC cases (Figures 3(e)–3(h)). Third, KRT8 expression was found to be associated with the overall survival of PDAC patients in two distinct cohorts (Figures 4(a) and 4(b)).

An association between inflammatory lesions and KRT8 expression was also found in a study on lung tissue injury, regeneration, and fibrosis [17]. Notably, despite possibly different molecular mechanisms, this process more or less resembles the development of pancreatic fibrosis and pancreatic dysfunction. Recent studies of pancreatic diseases also focused on the expression of KRT8. Zhong and Omary found reactive upregulation of KRT8 in caerulein-induced mouse pancreatitis models [19]. Moreover, they demonstrated that in benign lesions, KRT8 might serve protective roles, indicating that the function of KRT8 might change as the disease developed from pancreatitis to PDAC, accompanied by the change of KRT8 expression (Figure 6(e)) [19]. Even so, studies on KRT8 in pancreatitis have mostly focused on mutations and sequence variants [45–47]. In summary, 5%-10% of the pancreatitis patients harboured different types of KRT8 mutations caused by single nucleotide variant (SNV), such as glycine-to-cysteine mutation at position 61 and alanine-to-valine mutation at position 358 [46, 47]. Isoleucine-to-threonine mutation at position 321 was found in some Chinese children who suffered from recurrent pancreatitis [45]. Pistoni et al. combined these findings and pointed out that the reason might be that certain types of gene polymorphisms could promote KRT8 expression [18]. Our study agreed with the findings above. We not only provide more evidence of KRT8 upregulation in pancreatitis and PDAC (Figures 6(a)–6(g)) but also demonstrate the role of KRT8 as an unfavourable prognostic and diagnostic biomarker (Figures 3(e)–3(h) and 4(a)–4(c)).

The function of KRT8 in PDAC was predicted by protein-protein interaction analysis, correlation analysis, and GO analysis. Two hundred and thirteen DEGs were found to correlate with KRT8 by the Pearson method (Figures 5(a) and 5(b)). The result of enrichment analysis revealed that nearly 7% of these DEGs could play roles in regulating the migration ability of PDAC cells (Figure 5(c), Table S6). Besides, some important cell membrane proteins were found to potentially bind to KRT8 (Figure S5). Three correlated DEGs were validated by RT-qPCR, and we found that KRT18 and YWHAZ could be regulated by KRT8 (Figure 8(b)). Sun et al. analysed hepatocellular carcinoma data with a similar method, but the function prediction was not discussed in detail [48]. Here we suggest that correlation analysis, enrichment analysis, and protein-protein interaction analysis should be integrated to improve the accuracy of predicting the function of a single gene. Using this method, we found that KRT8 might be involved in the regulation of migration and cell viability. Knockdown of KRT8 impaired the migration ability of two PDAC cell lines in which KRT8 was upregulated (Figures 7(a)–7(c) and 7(e)), which was similar to what Stanton et al. observed in breast cancer [49]. The proliferation of PDAC cells was also inhibited in vivo and in vitro (Figures 7(d) and 7(h)). KRT8 knockdown led to cell cycle arrest in G2/M phase and induced apoptosis (Figures 7(f) and 7(g)). By Western blot, we found that KRT8 knockdown could regulate the expression of EMT markers and apoptosis mediators (Figure 8(a)). The conclusion could be further validated by the clinicopathological data. We found KRT8 was correlated with T classification of PDAC, a parameter that is strongly associated with the degree of malignancy, excessive proliferation, and invasiveness (Figure 4(d) and Table 2). Besides, it is worth noting that our conclusion about the effect of KRT8 on migration agreed with that of a study by Fang et al. in gastric cancer and conflicted with the findings of studies by Yee et al. in prostate cancer and Li et al. in lung cancer [10, 14, 16]. The reason might be as follows: First, the function of KRT8 might be different in distinct types of tissue. Second, the effect on migration might be influenced by inflammation-associated molecular mechanisms independent of the EMT pathway [50]. Third, posttranslational modification might change the function of the KRT8 protein, which has not been considered in most studies [9].

Our study has several advantages. We applied bioinformatic methods to identify biomarkers for pancreatitis and PDAC. A total of 102 mouse samples and 504 human samples were included for analysis. Our findings could be used for mechanism research on the development of pancreatitis. In addition, KRT8 can be utilized not only as an IHC marker but also as a serum marker because KRT8 can be detected in peripheral blood [51]. The diagnostic value of KRT8 was assessed by ROC curves. We found that KRT8 could be used for the differentiation between PDAC and normal cases. However, in certain datasets (GSE62452 and GSE71729), the diagnostic value was not as high as the others, which could be explained by relatively small sample size. Nevertheless, there are still some limitations. First, we failed to obtain human AP samples, although CP samples are more useful for PDAC research. Secondly, due to the lack of related materials, other types of pancreatic tumours were not analysed. Third, despite the finding of KRT8 upregulation in pathological tissue, the underlying molecular mechanism remains to be further studied.

## 5. Conclusions

KRT8 is upregulated in pancreatitis and PDAC in an incremental manner, indicating that KRT8 can play a role in the development from pancreatitis to PDAC. KRT8 is an inflammatory molecule and can serve as an unfavourable prognostic marker that has a negative impact on the prognosis of PDAC patients. KRT8 also has diagnostic value in distinguishing PDAC cases from normal cases. In addition, KRT8 is an important regulator of the migration and viability of PDAC cells. More studies are needed for further validation from the perspective of precision and individualized medicine.

## Figures and Tables

**Table 1:** Cox regression analyses of clinicopathological features associated with PDAC patient survival in TCGA.

Parameter	Univariate analysis			Multivariate analysis		
	HR	HR 95% CI	*P* value	HR	HR 95% CI	*P* value
Age	1.403	0.814-2.416	0.121			
Histologic grade	1.424	0.919–2.205	0.113			
N classification	2.180	1.283–3.706	*0.004*	1.861	1.050-3.299	*0.033*
T classification	1.838	0.948–3.562	0.071			
Gender	0.781	0.514–1.187	0.247			
Stage	0.802	0.253–2.546	0.708			
KRT8 expression	1.394	1.098-1.771	*0.006*	1.364	1.055-1.765	*0.018*

HR: hazard ratio; CI: confidence interval. Italicized value indicates *P* < 0.05.

**Table 2:** Association of KRT8 expression with clinicopathological features in PDAC patients from TCGA.

Variables	Case (number, %)	KRT8 expression level (number, %)		*P* value
		High expression (*N* = 83)	Low expression (*N* = 84)	
Age (years)	≤55 (19.8)	17 (51.5)	16 (48.5)	0.848
	>55 (80.2)	66 (49.3)	68 (50.7)	
Histologic grade	G1+G2 (70.1)	54 (46.2)	63 (53.8)	0.161
	G3+G4 (29.9)	29 (58.0)	21 (42.0)	
N classification	N0 (28.1)	22 (46.8)	25 (53.2)	0.640
	N1 (71.9)	61 (50.8)	59 (49.2)	
T classification	T1+T2 (16.8)	8 (28.6)	20 (71.4)	*0.014*
	T3+T4 (83.2)	75 (54.0)	64 (46.0)	
Gender	Female (45.5)	34 (44.7)	42 (55.3)	0.241
	Male (54.5)	49 (53.8)	42 (46.2)	
Stage	Stage I+II (95.8)	81 (50.6)	79 (49.4)	0.253
	Stage III+IV (4.2)	2 (28.6)	5 (71.4)	

Italicized value indicates *P* < 0.05.

## Data Availability

The microarrays analysed in the current study are available in online databases (GEO database and UCSC Xena database). The datasets analysed during the current study are available from the corresponding author on reasonable request.

## Supplementary Materials

Supplementary Materials Additional file 1: Figure S1. Figure 1: illustration of the work flow diagram. Additional file 2: Figure S2: presentation of the DEGs of murine AP models. The result was visualized by hierarchical cluster heatmaps. (A) GSE3644. (B) GSE109227. (C) GSE121038. The gradual change from red to blue represents the changes of gene expression from high to low. The white color represents no difference in gene expression. (D) Venn diagrams for the intersection of DEGs among different datasets. (E) GO analysis for the DEGs in the intersection. (F) KEGG analysis for the DEGs in the intersection. Additional file 3: Figure S3: presentation of the DEGs of murine CP models. The result was visualized by hierarchical cluster heatmaps. (A) Jackson mice group in GSE41418. (B) Harlan mice group in GSE41418. (C) Venn diagrams for the intersection of DEGs between different datasets. (D) GO analysis for the DEGs in the intersection. (E) KEGG analysis for the DEGs in the intersection. Additional file 4: Figure S4: presentation of the expression of some genes that were not influenced by pancreatitis in GSE40895. Additional file 5: Figure S5: the interaction between KRT8 and other genes in correlation analysis. Additional file 6: Figure S6: illustration of the genes involved in different pathways retrieved in PathCards database. The gradual change from red to blue represents the changes of relevance score from high to low. The white colour represents the genes that have no significant connection with the pathways. Additional file 7: Figure S7: presentation of the genes with the top three relevance scores from high to low in the pathways involved in inflammation process provided by PathCards database. (A) Innate immune system. (B) Interferon. (C) Interleukin. (D) B cell receptor signaling pathway. (E) NF-*κ*B signaling pathway. ^∗∗∗^*P* < 0.001. ^∗∗^*P* < 0.01. Additional file 8: Figure S8: presentation of the genes with the top three relevance scores from high to low in the pathways involved in cell viability regulation provided by PathCards database. (A) Akt signaling pathway. (B) MAPK signaling pathway. (C) Apoptosis. ^∗∗∗^*P* < 0.001. Additional file 9: Figure S9: representative images of the wound healing assay. Red bar, 100 *μ*m. Additional file 10: Figure S10: representative images of the positive control group in apoptosis analysis. Additional file 11: Figure S11: survival analysis of KRT18 with TCGA and GSE62452 cohorts. Additional file 12: Table S1: details of the microarrays downloaded from GEO database. Additional file 13: Table S2: the results of GO analysis of the AP-associated DEGs. Additional file 14: Table S3: the results of KEGG analysis of the AP-associated DEGs. Additional file 15: Table S4: the results of GO analysis of the CP-associated DEGs. Additional file 16: Table S5: the results of KEGG analysis of the CP-associated DEGs. Additional file 17: Table S6: the results of GO analysis of the KRT8-correlated DEGs. Additional file 18: Table S7: presentation of common KRT8-correlated DEGs, relevance score of each pathway retrieved in PathCards database. Additional file 19: Table S8: detailed information of the patients mentioned in this study.
