# Supplementary material for: Keratin 8 Is an Inflammation-Induced and Prognosis-Related Marker for Pancreatic Adenocarcinoma
Source: Dis Markers. 2022 Jul 27;2022:8159537. doi: 10.1155/2022/8159537 (PMC9359862; doi:10.1155/2022/8159537)
Supplement: Supplementary Materials — Additional file 1: Figure S1. Figure 1: illustration of the work flow diagram. Additional file 2: Figure S2: presentation of the DEGs of murine AP models. The result was visualized by hierarchical cluster heatmaps. (A) GSE3644. (B) GSE109227. (C) GSE121038. The gradual change from red to blue represents the changes of gene expression from high to low. The white color represents no difference in gene expression. (D) Venn diagrams for the intersection of DEGs among different datasets. (E) GO analysis for the DEGs in the intersection. (F) KEGG analysis for the DEGs in the intersection. Additional file 3: Figure S3: presentation of the DEGs of murine CP models. The result was visualized by hierarchical cluster heatmaps. (A) Jackson mice group in GSE41418. (B) Harlan mice group in GSE41418. (C) Venn diagrams for the intersection of DEGs between different datasets. (D) GO analysis for the DEGs in the intersection. (E) KEGG analysis for the DEGs in the intersection. Additional file 4: Figure S4: presentation of the expression of some genes that were not influenced by pancreatitis in GSE40895. Additional file 5: Figure S5: the interaction between KRT8 and other genes in correlation analysis. Additional file 6: Figure S6: illustration of the genes involved in different pathways retrieved in PathCards database. The gradual change from red to blue represents the changes of relevance score from high to low. The white colour represents the genes that have no significant connection with the pathways. Additional file 7: Figure S7: presentation of the genes with the top three relevance scores from high to low in the pathways involved in inflammation process provided by PathCards database. (A) Innate immune system. (B) Interferon. (C) Interleukin. (D) B cell receptor signaling pathway. (E) NF-κB signaling pathway. ∗∗∗P < 0.001. ∗∗P < 0.01. Additional file 8: Figure S8: presentation of the genes with the top three relevance scores from high to low in the pathways involved in cell via [file 8159537.f1.zip › Table S1-S6 (1).docx]

Table S1. Details of the microarrays downloaded from GEO database.

Microarrays of murine pancreatitis models

| ID | Platform | Control | Pancreatitis |
| --- | --- | --- | --- |
| GSE3644 | GPL339 | 3 | 3 |
| GSE40895 | GPL6246 | 12 | 9 |
| GSE41418 | GPL1261 | 6 | 6 |
| GSE65146 | GPL6246 | 5 | 39 |
| GSE109227 | GPL6246 | 5 | 6 |
| GSE121038 | GPL10787 | 4 | 4 |
| Total |  | 35 | 67 |

Microarrays of Human PDAC and pancreas

| ID | Platform | Pancreas | PDAC |
| --- | --- | --- | --- |
| GSE15471 | GPL570 | 39 | 39 |
| GSE16515 | GPL570 | 16 | 36 |
| GSE32676 | GPL570 | 7 | 25 |
| GSE62452 | GPL6244 | 61 | 69 |
| GSE71729 | GPL20769 | 46 | 145 |
| GSE71989 | GPL570 | 8 | 13 |
| Total |  | 177 | 327 |

Table S2. The results of GO analysis of the AP-associated DEGs.

| Category | ID | Term | Count | adj.P | Genes |
| --- | --- | --- | --- | --- | --- |
| BP | GO:0070527 | Platelet aggregation | 7 | 0.000495009 | CSRP1, MYH9, SLC7A11, CLIC1, ACTB, VCL, ACTG1 |
| BP | GO:0051591 | Response to cAMP | 6 | 0.025355053 | JUN, JUND, MAT2A, DUSP1, CREM, RELA |
| BP | GO:1990440 | Positive regulation of transcription from RNA polymerase II promoter in response to endoplasmic reticulum stress | 4 | 0.025505101 | CEBPB, CREB3L1, ATF3, ATF4 |
| CC | GO:0005737 | Cytoplasm | 98 | 5.5732E-15 | CARS, TES, PRDM4, PREP, SAT1, ACTB, ACTG1, RGS2, TUBB6, SLK, CDH1, CAPN2, CHAC1, CCNL1, PHLDA1, IER2, MAP2K3, CAST, TPM4, FBXW11, ACTN1, IFRD1, KRT8, ACSL4, ACTN4, GTPBP4, ARC, KCTD10, TRIB1, RIN1, SQSTM1, VCL, ATF4, PARM1, HSPA4L, CREM, NMD3, IQGAP1, RTN4, FAM83H, PACSIN3, RALGDS, HSPA9, EGR1, CHKA, GOT1, RBPMS, DSTN, RCAN1, GJB2, APPBP2, SERPINB6A, RCAN3, CPEB2, FERMT2, KDM5B, CDKN1A, CLIC4, SRXN1, HSPB8, PTPN23, DUSP16, PCMTD2, GYS1, CCKAR, CLIC1, YWHAH, TRP53INP1, DSP, HSP90AA1, DUSP1, RIPK4, DYNLL2, RBL2, CLDN10, TUBB2A, CDC42EP5, MYH9, GARS, ARF6, CEBPB, CTDP1, SRF, NEDD9, TOB1, RELA, PPP2CA, LMAN1, NCDN, NOP58, GCH1, TUBB4B, KLF6, KRT18, ZYX, KRAS, EPPK1, TJP2 |
| CC | GO:0005925 | Focal adhesion | 19 | 4.95658E-08 | HSPA9, TES, TPM4, ACTN1, RRAS2, ACTN4, IQGAP1, ACTB, ACTG1, CSRP1, CDH1, ZYX, CAPN2, MYH9, KRAS, ITGA6, FERMT2, VCL, ARF6 |
| CC | GO:0005634 | Nucleus | 79 | 9.69684E-08 | KDM5B, CDKN1A, CLIC4, TES, PRDM4, HSPB8, PREP, PTPN23, DUSP16, ACTG1, RGS2, TUBB6, CSRP1, CREB3L1, CAPN2, CCNL1, CLIC1, PHLDA1, IER2, TRP53INP1, IER3, DSP, TGIF1, CAST, HSP90AA1, FBXW11, DUSP1, ACTN1, IFRD1, KRT8, H2AFV, ACTN4, DYNLL2, KLF15, GTPBP4, RBL2, TUBB2A, KCTD10, MYH9, TRIB3, TRIB1, SQSTM1, ATF3, ATF4, CEBPB, PARM1, CTDP1, SRF, CEBPG, HSPA4L, CREM, NEDD9, NMD3, IQGAP1, TOB1, RELA, PPP2CA, NFIL3, RALGDS, HSPA9, EGR1, NOP58, JUN, JUND, GCH1, RBPMS, GOT1, NFYB, TUBB4B, RCAN1, KLF7, KLF6, KRT18, APPBP2, ZYX, UBA3, CPEB2, FERMT2, TJP2 |
| CC | GO:0005913 | Cell-cell adherens junction | 16 | 5.06673E-07 | CAST, TES, IQGAP1, PVR, RTN4, SLK, KRT18, CDH1, ZYX, EFHD2, MYH9, TAGLN2, ITGA6, CLIC1, VCL, TJP2 |
| CC | GO:0001725 | Stress fiber | 9 | 1.1608E-06 | TPM4, ACTN1, ZYX, MYH9, ACTN4, ACTB, MYL12A, VCL, FERMT2 |
| CC | GO:0070062 | Extracellular exosome | 43 | 1.82966E-05 | DDR1, CLIC4, IQGAP1, PTPN23, ACTB, MYL12A, RTN4, ACTG1, PPP2CA, LMAN1, TUBB6, SLK, CSRP1, CDH1, EPCAM, CAPN2, PACSIN3, CLIC1, YWHAH, HSPA9, DSP, HSP90AA1, TPM4, GOT1, ACTN1, KRT8, H2AFV, RRAS2, DSTN, ACSL4, ACTN4, TUBB4B, RBL2, TUBB2A, KRT18, SERPINB6A, MYH9, GARS, TAGLN2, PLP2, SQSTM1, VCL, ARF6 |
| CC | GO:0005856 | Cytoskeleton | 25 | 4.14273E-05 | CTDP1, NEDD9, SLC7A11, PTPN23, ACTB, ACTG1, FAM83H, PPP2CA, TUBB6, DSP, TPM4, ACTN1, TUBB4B, DYNLL2, ARC, TUBB2A, APPBP2, CDC42EP5, ZYX, MYH9, EPPK1, RIN1, FERMT2, VCL, ATF4 |
| CC | GO:0005911 | Cell-cell junction | 11 | 5.39074E-05 | DSP, OCLN, CLIC4, CDH1, ACTN1, KRT8, EPPK1, ACTN4, IQGAP1, VCL, TJP2 |
| CC | GO:0016327 | Apicolateral plasma membrane | 5 | 0.000330977 | OCLN, CLDN8, CLDN7, KRT8, EPPK1 |
| CC | GO:0015629 | Actin cytoskeleton | 10 | 0.000331327 | ARC, CTDP1, CSRP1, CDH1, MYH9, DSTN, ACTN4, IQGAP1, VCL, ACTG1 |
| CC | GO:0016328 | Lateral plasma membrane | 6 | 0.00083156 | OCLN, GJB2, EPCAM, CDH1, CLDN7, IQGAP1 |
| CC | GO:0005829 | Cytosol | 29 | 0.001138952 | CDKN1A, CLIC4, SRXN1, HSPA4L, PREP, SAT1, DUSP16, ACTB, RELA, ACTG1, PPP2CA, RGS2, MAT2A, CAPN2, NCDN, CHAC1, RALGDS, YWHAH, TRP53INP1, CAST, MAP2K3, JUN, HSP90AA1, GCH1, GOT1, FBXW11, MTHFR, UBA3, MYH9 |
| CC | GO:0030863 | Cortical cytoskeleton | 5 | 0.001138952 | TPM4, ACTN1, MYH9, ACTN4, ACTB |
| CC | GO:0030054 | Cell junction | 16 | 0.003302721 | DSP, TES, ACTN1, ACTN4, CLDN10, OCLN, GJB2, ARC, CDH1, EPCAM, CLDN8, CLDN7, ZYX, FERMT2, VCL, TJP2 |
| CC | GO:0043234 | Protein complex | 14 | 0.008942096 | CDKN1A, HSP90AA1, TES, JUND, GCH1, ACTN4, IQGAP1, ACTB, MYL12A, RTN4, RELA, SERPINB6A, MYH9, VCL |
| CC | GO:0016323 | Basolateral plasma membrane | 8 | 0.00908713 | DDR1, DSP, HSP90AA1, EPCAM, CDH1, CLDN8, CLDN7, ITGA6 |
| CC | GO:0005903 | Brush border | 5 | 0.028985058 | ACTN1, MYH9, ACTN4, RALGDS, VCL |
| CC | GO:0005923 | Bicellular tight junction | 6 | 0.030271706 | CLDN10, OCLN, EPCAM, CLDN8, CLDN7, TJP2 |
| CC | GO:0043209 | Myelin sheath | 7 | 0.030271706 | HSPA9, HSP90AA1, TUBB4B, ACTB, RTN4, ARF6, ACTG1 |
| CC | GO:0005916 | Fascia adherens | 3 | 0.040261371 | DSP, ACTN1, VCL |
| CC | GO:0001726 | Ruffle | 5 | 0.049962787 | TNFRSF12A, ACTN1, MYH9, IQGAP1, ARF6 |
| MF | GO:0005515 | Protein binding | 70 | 1.563E-08 | CDKN1A, HSPB8, PVR, DUSP16, ACTB, GYS1, RGS2, SLK, CSRP1, CDH1, EFHD2, SOSTDC1, LRRFIP2, CCNL1, IER3, YWHAH, MAP2K3, DSP, TGIF1, HSP90AA1, TNFRSF12A, FBXW11, ACTN1, KRT8, RRAS2, ACTN4, DYNLL2, KLF15, RBL2, CDC42EP5, KCTD10, CLDN8, CLDN7, MYH9, GARS, ITGA6, TRIB3, SQSTM1, VCL, ATF4, ARF6, CEBPB, CTDP1, SRF, CEBPG, HSPA4L, CREM, IQGAP1, TOB1, RTN4, RELA, PPP2CA, NFIL3, PACSIN3, NCDN, RALGDS, HSPA9, JUN, JUND, GJB2, OCLN, KRT18, APPBP2, SERPINB6A, ZYX, UBA3, KRAS, CPEB2, LNX2, TJP2 |
| MF | GO:0098641 | Cadherin binding involved in cell-cell adhesion | 15 | 1.04891E-05 | CAST, TES, IQGAP1, RTN4, SLK, KRT18, CDH1, EPCAM, EFHD2, MYH9, TAGLN2, ITGA6, CLIC1, VCL, TJP2 |
| MF | GO:0032403 | Protein complex binding | 13 | 0.003878626 | CLIC4, CDKN1A, NFYB, KRT8, MTHFR, ACTN4, IQGAP1, RTN4, RELA, PPP2CA, EPCAM, KRAS, ITGA6 |
| MF | GO:0044212 | Transcription regulatory region DNA binding | 10 | 0.009780288 | EGR1, CEBPB, JUN, JUND, NFYB, CREB3L1, KLF15, RELA, ATF3, ATF4 |
| MF | GO:0042803 | Protein homodimerization activity | 17 | 0.03852999 | CARS, CEBPB, JUN, HSP90AA1, GCH1, RBPMS, CHKA, SRF, ACTN1, CEBPG, ACTN4, PVR, RELA, SLK, MYH9, SQSTM1, ATF3 |
| MF | GO:0000166 | Nucleotide binding | 30 | 0.03852999 | CARS, DDR1, SRXN1, HSPA4L, ACTB, ACTG1, TUBB6, SLK, MAT2A, SEPHS2, MAP2K3, HSPA9, HSP90AA1, GCH1, RBPMS, CHKA, RIPK4, RRAS2, ACSL4, TUBB4B, GTPBP4, RCAN1, TUBB2A, RCAN3, UBA3, MYH9, KRAS, GARS, CPEB2, ARF6 |
| MF | GO:0019901 | Protein kinase binding | 12 | 0.03852999 | FAM83H, PPP2CA, MAP2K3, GYS1, HSP90AA1, TRIB3, IQGAP1, PTPN23, CCNL1, SQSTM1, RELA, ACTB |

Table S3. The results of KEGG analysis of the AP-associated DEGs.

| ID | Term | Count | adj.P | Genes |
| --- | --- | --- | --- | --- |
| mmu04530 | Tight junction | 12 | 6.94199E-07 | PPP2CA, CLDN10, OCLN, CLDN8, ACTN1, CLDN7, MYH9, ACTN4, ACTB, MYL12A, TJP2, ACTG1 |
| mmu04670 | Leukocyte transendothelial migration | 10 | 0.000537896 | CLDN10, OCLN, CLDN8, ACTN1, CLDN7, ACTN4, ACTB, MYL12A, VCL, ACTG1 |
| mmu04520 | Adherens junction | 7 | 0.008060921 | CDH1, ACTN1, ACTN4, IQGAP1, ACTB, VCL, ACTG1 |
| mmu05203 | Viral carcinogenesis | 11 | 0.008569338 | RBL2, JUN, CDKN1A, SRF, CREB3L1, ACTN1, KRAS, ACTN4, RELA, YWHAH, ATF4 |
| mmu05166 | HTLV-I infection | 12 | 0.008569338 | EGR1, JUN, CDKN1A, NFYB, SRF, CREM, RRAS2, KRAS, RELA, ATF3, ATF4, TRP53INP1 |
| mmu04510 | Focal adhesion | 10 | 0.014114524 | JUN, ACTN1, ZYX, CAPN2, ITGA6, ACTN4, ACTB, MYL12A, VCL, ACTG1 |
| mmu04810 | Regulation of actin cytoskeleton | 10 | 0.014870907 | ACTN1, RRAS2, ITGA6, KRAS, ACTN4, IQGAP1, ACTB, MYL12A, VCL, ACTG1 |
| mmu05160 | Hepatitis C | 8 | 0.01674839 | PPP2CA, CLDN10, OCLN, CDKN1A, CLDN8, CLDN7, KRAS, RELA |
| mmu04151 | PI3K-Akt signaling pathway | 12 | 0.03288161 | RBL2, PPP2CA, GYS1, CDKN1A, HSP90AA1, CREB3L1, ITGA6, KRAS, OSMR, RELA, YWHAH, ATF4 |
| mmu04010 | MAPK signaling pathway | 10 | 0.03288161 | MAP2K3, JUN, JUND, DUSP1, SRF, RRAS2, KRAS, DUSP16, RELA, ATF4 |
| mmu05215 | Prostate cancer | 6 | 0.044353388 | CDKN1A, HSP90AA1, CREB3L1, KRAS, RELA, ATF4 |

Table S4. The results of GO analysis of the CP-associated DEGs.

| Category | ID | Term | Count | adj.P | Genes |
| --- | --- | --- | --- | --- | --- |
| BP | GO:0044822 | Poly(A) RNA binding | 137 | 6.01218E-13 | TCERG1, SRP19, DBR1, RPL3, TFRC, ZAK, H2-K1, PPAN, PEBP1, NUCKS1, FBL, LGALS3, SNRPD1, MAGOH, SNIP1, QK, SNRPD3, KPNA2, PDIA3, FNDC3B, DDX10, DNTTIP2, UTP11L, ILF2, PDIA4, FAM133B, DDX39B, PSPC1, SARNP, MRTO4, XRN2, SRSF2, SRSF3, ERH, KCTD12, EIF1A, SRSF7, H2-D1, SNRPB, SLBP, RPS19BP1, CSTF3, RPN1, CSTF2, ZCCHC9, ISG20L2, NMD3, CORO1A, MRPL13, GSPT1, RTN4, MNDAL, STIP1, HNRNPDL, PRDX1, RRS1, BRIX1, LLPH, RBM12, HSPA9, PNPT1, ZRANB2, UBE2I, RBPMS, LARP7, MEX3C, EIF2S1, PARP12, MANF, BST2, CCT6A, HNRNPM, MEX3B, HNRNPC, NOL11, LSM14A, TOP2A, COL14A1, CCDC47, WDR3, CELF2, HMGB2, WDR43, SYNCRIP, CCT3, HSP90AA1, ANXA2, PRMT1, NCBP2, SPATS2, ZFR, DDX50, CKAP4, GNL3, NME1, SUPV3L1, MAPRE1, SKIV2L2, AHNAK, RPL12, H2-Q7, SRSF1, HSPD1, PURB, PABPN1, FAM120C, EXOSC9, TRA2B, TRA2A, EIF4H, RPL13, HNRNPA1, SRSF10, HNRNPA0, NOP56, PTCD3, NOP58, DUT, TAF15, MYEF2, IFI204, NIFK, MDH2, IFI205, CNBP, FAM46A, NAP1L1, SSRP1, HSPE1, BICC1, NAP1L4, KRT18, TRMT6, FAM98B, SSBP1, TARDBP, RAN |
| BP | GO:0036094 | Small molecule binding | 15 | 6.82235E-07 | GM2083, MUP10, AMBP, MUP15, MUP16, MUP7, MUP1, MUP12, MUP13, MUP2, MUP14, MUP8, MUP19, MUP9, LCN2 |
| BP | GO:0003723 | RNA binding | 88 | 5.15648E-06 | SRP19, CELF2, POP4, NUDT1, HMGB3, BRCA1, NUDT5, NUDT4, IFIH1, FBL, SYNCRIP, METTL14, XPO1, SNRPD1, PAPOLG, MAGOH, QK, SNRPD3, ALYREF2, TRIM21, OASL2, RBM17, MORC3, SNRPN, KDM2B, DIS3, NCBP2, ZFR, DDX10, TRNT1, DDX50, ILF2, MARF1, RBMXL1, DDX39B, PSPC1, LACTB2, SRSF2, SNRPG, SRSF3, SNRPF, SNRPC, EIF1A, SRSF7, SKIV2L2, SNRPB, RBMS3, SLBP, RNASEL, RNASEH2A, RNMT, RPL12, CSTF2, SRSF1, PURB, PABPN1, HNRNPDL, EXOSC9, TRA2B, EXOSC8, TRA2A, EIF4H, RPL13, SNRPB2, BRIX1, HNRNPA1, RBM12, SRSF10, PAIP1, HNRNPA0, PTCD3, PNPT1, ZRANB2, RBPMS, NIFK, ALYREF, LARP7, MEX3C, EIF2S1, BICC1, HNRNPM, CNOT6, MEX3B, CNOT7, IMPDH2, HNRNPC, TARDBP, EZH2 |
| BP | GO:0019899 | Enzyme binding | 53 | 7.68424E-06 | TOP2A, YWHAB, PEBP1, BRCA1, CDC20, HIST1H2AE, COTL1, HIST1H2AD, BANF1, HIST1H2AG, SNRPD3, LGALS9, HIST1H2AC, HIST1H2AB, RFC5, HIST1H2AO, HIST1H2AN, RFC4, H2AFY, UGT1A1, HIST1H2AI, RFC2, PRMT1, H2AFX, DYNLL1, HIST1H2AP, NME1, AR, PLSCR1, ADORA2A, BIRC5, SLC25A4, PPID, MCM2, NOTCH2, PCNA, RPS19BP1, HDAC1, BCL10, THY1, HIF1A, UNG, ZNHIT6, DYM, HSPA9, UBE2I, CBX3, STAT1, PTGES3, EPHX1, RPA2, ALOX5AP, CALU |
| BP | GO:0005515 | Protein binding | 321 | 7.68424E-06 | CYFIP2, ATF2, PID1, SPARC, TFRC, PGAP2, ARL6IP1, CLDN2, CDC20, LGALS3, GJA1, ZFP281, PPP4R2, ALKBH1, PPP4R4, MYC, DPYSL3, CHORDC1, CHEK1, STMN1, MYB, AP1S2, PSMD1, FBXO5, SKP2, B2M, TRIM21, RNF111, IER3, TMPO, CDON, TIPIN, IL1R1, KRT8, KRT7, GTPBP2, QTRTD1, WDR77, AR, TIAM1, TWSG1, CLDN4, KCTD10, SARNP, XRN2, PSME1, KCTD12, NBL1, EPHA3, H2-D1, PFN2, IGSF5, BEX1, CTBP2, ANP32A, SDC2, CDCA8, VPS26A, BCL10, HIF1A, MTMR7, PLAC8, PKHD1, PRDX2, RRS1, APOE, STX4A, ABCA1, PLK4, H2-EB1, CADM1, BCL11A, GADD45A, ZWINT, CCT6A, EHD4, KITL, FERMT3, EZH2, CDKN1C, BTG3, NCAPG2, KRT20, ZFP809, IFIH1, TUBA1B, XPO1, CSRP2, CTSL, TMSB4X, BCL2A1A, CEP55, RAB8B, APOBEC1, CCT3, ANXA1, FCER1G, ANXA2, PRMT1, PARP2, ACTL6A, ATPIF1, TCF12, ANXA5, SERPINB9, DYNLL1, ZDHHC17, GNL3, PSMA3, RAD51D, MDFIC, PSMA1, RHOJ, GAS1, CD48, PLIN2, TAX1BP3, AIDA, MAPRE1, CRYAB, CD44, PCNA, ATL1, RPL12, AIF1, PTS, PURB, LMNA, CKB, PAIP1, ATAD1, CD74, NFYA, USP9X, TMEM176B, TMEM176A, RPA2, TTC8, KRT18, CAPZA1, CLCF1, CAPZA2, RLIM, TRIP13, TARDBP, FAM49B, ALDH1L1, NCF1, RIF1, CD81, CSE1L, LOXL1, AMOT, RGS2, CCND2, SLK, BMPER, BASP1, RUVBL1, ADORA1, COTL1, SNIP1, DENND5A, KPNA2, HMGN1, PDIA3, SCARA5, UBE2E3, LMO2, WNT5A, ADAM10, CYBB, CYBA, FRMD4B, ATP1B1, CASD1, RAB32, ACAP2, TYROBP, RBMXL1, MAF, BRMS1, LOX, STIM1, PSPC1, TBL1XR1, RAB34, S100A6, SRSF2, PLAA, FKBP7, GEMIN6, SRSF7, SNRPB, NOTCH2, FBN2, CBFB, PSMD14, RPS19BP1, HTRA3, SLC41A3, RHOBTB3, HTRA1, TOR1AIP2, FBLN1, NREP, CORO1A, RTN4, RAP1B, ATXN3, SYCE2, STIP1, CCNB1, BAG5, NDN, LLPH, ECT2, RAB6A, GRIA3, HSPA9, PRNP, CTLA2B, UBE2I, AUTS2, CBX3, RARRES2, EIF2S1, CDK7, DAB2, CNOT7, PTPRC, NASP, ATG16L1, CDK4, DNAJC10, CDK1, LCN2, UBA2, SLC26A6, NFE2L2, H2-AB1, FBN1, ZFP110, KDM5A, MCM7, YWHAB, SNAP23, SLC40A1, TRF, LRMP, TREM2, RND2, RND3, METTL14, CD1D1, CASP8, CAPZB, BLNK, EFHD2, PMEPA1, SKP1A, SRGN, RBM17, HSP90AA1, PPP1R12A, CHUK, SGIP1, H2AFX, TGFBR1, TGFBR2, CCNA2, RBL1, TFDP1, PSRC1, BIRC5, IRF8, CDH13, MCM4, NAA35, TRIM59, MCM6, SLC25A4, ITIH4, USP14, SEC23A, RGS19, HDAC1, UHRF1, VPS4B, POMP, THBS1, THBS4, EHBP1, CLEC3B, TTR, RBBP4, PABPN1, TDG, TRA2B, STOM, SNRPB2, E2F3, RRM1, RRM2, CMTM3, IFI204, EGF, NAP1L1, H2-AA, BICC1, DNAJA1, MFAP5, REST, RAD51, ALOX5AP, RGS10, CD9, BAX, VIM, PTPN2, RAN |
| BP | GO:0003697 | Single-stranded DNA binding | 19 | 0.000296542 | ANXA1, MCM7, PRIM1, CNBP, ALYREF, RPA1, RPA2, NUCKS1, NME1, PURB, RAD51D, RAD51, RPA3, MCM4, NUP35, MCM6, SSBP1, ALYREF2, HNRNPA1 |
| BP | GO:0042802 | Identical protein binding | 68 | 0.000433783 | TFRC, APIP, HJURP, CLDN2, PTPRG, ACTG1, SLK, CASP8, IMPA1, A730008H23RIK, EMILIN1, TOPBP1, SKP2, IER5, TRIM21, B2M, CTSC, HSP90AA1, APAF1, PRMT1, ANXA4, SFTPD, ZDHHC17, NME1, CLDN4, TYROBP, STIM1, ADORA2A, DDX39B, CLDN9, BIRC5, MCM6, MAPRE1, CRYAB, OAT, PCNA, UHRF1, ATL1, LYZ2, VPS4B, LYZ1, MGST1, FBLN1, BCL10, CORO1A, PTS, ATXN3, TTR, ZNHIT6, STOM, BNIP2, APOE, PLK4, PRNP, CBX3, STAT1, HPRT, RAD51, ATG16L1, 2310057M21RIK, BAX, ESYT2, HNRNPC, VIM, TRIP13, TARDBP, PICALM, MAD2L1 |
| BP | GO:0042803 | Protein homodimerization activity | 80 | 0.001036829 | TOP2A, UGT1A10, TFRC, CPQ, HEXB, PLEK, NUDT5, HHEX, SLK, IMPA1, NAMPT, BANF1, BCL2A1D, BCL2A1A, CTSE, BCL2A1B, SBF2, HSP90AA1, ANXA1, CHUK, UGT1A1, TESC, TCF12, ATPIF1, TPM1, ADAM10, MAT1A, DYNLL1, QTRTD1, UGT1A6A, DCK, UGT1A6B, SUPV3L1, RRAGA, MAF, S100A6, CDH13, BIRC5, CRYAB, NBL1, PAFAH1B2, CRP, C1QB, BHLHB9, CTBP2, TIMM9, VPS4B, H2-M3, MGST1, BCL10, TYMS, CORO1A, PTS, TDG, PRDX1, PDGFC, UGT1A5, AGR2, STOM, APOE, UGT1A2, ECT2, UGT1A9, S100A10, SLC16A1, AMBP, CADM1, RBPMS, CAV2, STAT1, HPRT, EXT1, GGCT, GALE, FAP, ALOX5AP, LCN2, NUP35, BAX, MAD2L1 |
| BP | GO:0000166 | Nucleotide binding | 162 | 0.001106458 | ZAK, PEBP1, TTF2, SMC2, ACTG1, SLK, TUBB5, PPIP5K2, PAPOLG, CHEK1, RPS6KA1, RUVBL1, PIP4K2A, LIG1, UBE2E3, DDX10, TRNT1, MAT1A, GTPBP2, ATAD3A, DGKZ, RAB32, RRAGA, MARF1, RAB31, RBMXL1, DDX39B, PRKD3, PSPC1, RAB34, SRSF2, SRSF3, SRSF7, EPHA3, ATP6V1A, CSTF2, RHOBTB3, AK7, GSPT1, RAP1B, HNRNPDL, D5ERTD579E, RAB6A, RBM12, PCK2, ABCA1, PLK4, HSPA9, UBE2I, RBPMS, UBE2C, ALYREF, ABCA9, LARP7, KTI12, CDC7, CDC6, HPRT, PARP14, DCLK1, CCT6A, ICK, HNRNPM, CDK7, EHD4, CDK4, UBE2S, CDK1, UBA2, SRPRB, RAD17, CDK12, HNRNPC, TOP2A, MCM7, CHD9, CELF2, RND2, RND3, IFIH1, SYNCRIP, TUBA1B, TUBA1A, RARS, REV3L, PBK, ALYREF2, OASL2, RAB8B, CCT3, RFC5, RBM17, HSP90AA1, RFC4, APAF1, CHUK, RFC2, RMI1, NCBP2, DDX50, DCK, TGFBR1, TGFBR2, GNL3, NME1, RAP2C, SUPV3L1, RAD51D, TUBB2A, DALRD3, RHOJ, MCM3, MCM4, MCM5, KIF2C, MCM6, RHOV, KIFC3, SKIV2L2, DDR2, MCM2, RBMS3, TOR4A, DTYMK, RNASEL, RALB, ATL1, VPS4B, SRSF1, TYMS, HSPA13, ADCY7, HSPD1, NRAS, PABPN1, TRA2B, TRA2A, EIF4H, SNRPB2, CKB, HNRNPA1, SRSF10, MAP4K4, HNRNPA0, ATAD1, RRM1, TAF15, MYEF2, NIFK, ATAD2, TUBE1, TUBB4B, RAD51, AXL, IMPDH2, TAOK1, STK17B, NUP35, ATP13A2, TRIP13, TARDBP, RAN |
| BP | GO:0051117 | ATPase binding | 18 | 0.001774929 | ABCA1, NOP58, RALB, TAF9, ATPIF1, TOR1AIP2, ATP1B1, ATOX1, FBL, DNAJB1, ATXN3, AR, ZNHIT6, DNAJC10, RUVBL1, FXYD1, SNURF, RAB6A |
| BP | GO:0015020 | Glucuronosyltransferase activity | 10 | 0.00301595 | EXT1, UGT1A10, UGT1A1, UGT8A, UGT1A5, UGT1A2, UGT1A9, UGT1A6A, UGT1A6B, UGT1A7C |
| BP | GO:0001968 | Fibronectin binding | 10 | 0.00301595 | CCDC80, CTSK, MMP2, FBLN1, ITGAV, HSD17B12, THBS1, CTSS, THBS4, CTGF |
| BP | GO:0003678 | DNA helicase activity | 8 | 0.004133511 | SUPV3L1, MCM7, RUVBL1, MCM3, MCM4, MCM5, MCM6, MCM2 |
| BP | GO:0098641 | Cadherin binding involved in cell-cell adhesion | 35 | 0.005387035 | AHNAK, YWHAB, TWF1, CAPG, RTN4, DNAJB1, CCNB2, LDHA, SLK, CAPZB, EPCAM, RARS, PRDX1, RUVBL1, EFHD2, EIF4H, CLIC1, ARGLU1, SNX5, TMPO, NOP56, RANBP1, ANXA1, ANXA2, STAT1, ANLN, KRT18, FMNL2, EHD4, CAPZA1, ESYT2, MAPRE1, PHLDB2, RAN, PICALM |
| BP | GO:0003689 | DNA clamp loader activity | 6 | 0.005825165 | RFC5, RFC3, RFC4, RFC2, DSCC1, RAD17 |
| BP | GO:0046982 | Protein heterodimerization activity | 54 | 0.005863773 | TOP2A, ATF2, UGT1A10, HEXB, HEXA, H2-K1, SMC2, CASP8, MYC, ADORA1, ITGAV, BCL2A1D, BCL2A1A, BCL2A1B, SOX4, CHUK, H2AFY, KRT4, UGT1A1, HIST1H2AH, TCF12, H2AFX, CYBB, CYBA, QTRTD1, UGT1A6A, TGFBR1, UGT1A6B, RRAGA, MAF, ADORA2A, PAFAH1B2, H2-D1, H2-Q7, BCL10, HIF1A, CENPA, HSPD1, TTR, UGT1A5, UGT1A2, UGT1A9, CENPW, CAV2, H2-AA, EXT1, AXL, CLCF1, ALOX5AP, UBA2, BAX, SAE1, NFE2L2, H2-AB1 |
| BP | GO:0008201 | Heparin binding | 23 | 0.006439928 | TMEM184A, POSTN, MMP7, SERPINE2, CFH, PCOLCE2, PCOLCE, PTN, HMGB1, HSD17B12, THBS1, FSTL1, THBS4, CTGF, ADAMTS5, CLEC3B, PTPRC, CCL8, CCDC80, GM21596, APOE, FBN1, PF4 |
| BP | GO:0016787 | Hydrolase activity | 127 | 0.009048246 | DBR1, POP4, NUDT1, TTF2, NUDT5, CNDP2, NUDT4, RUVBL1, DPYSL3, DDX10, ADAM10, YOD1, DDX39B, PPA1, MTHFD2, LACTB2, PDE12, XRN2, PLBD1, PADI2, AMY1, PLA1A, ATP6V1A, RNASEH2A, PSMD14, LYZ2, HTRA3, CFI, LYZ1, RHOBTB3, ISG20L2, HTRA1, HTRA2, PLA2G7, MTM1, MTMR7, ATXN3, DPP7, RNF213, CLCA2, INPP5J, CLCA1, PNPT1, KLK1B24, EPHX1, CNOT6, CNOT7, PTPRC, PTPRA, PLCH1, CTRC, LGMN, MCM7, CHD9, CPQ, HEXB, HEXA, CTSZ, USP34, CTSS, PTPRG, IFIH1, SENP7, SCPEP1, ADAMTS5, CTSO, ADAMTS2, CASP8, IMPA1, CTSL, CES2G, CTSK, KLK1B5, CD38, CTSH, CPXM1, CTSE, ACP1, HGFAC, CTSC, CTSB, APOBEC1, MMP7, DIS3, MMP2, DUSP26, DDX50, YDJC, SUPV3L1, PSMA3, PSMA4, PSMA1, PTRH2, MCM3, MCM4, MCM5, MCM6, SMPDL3A, SKIV2L2, PAFAH1B2, MCM2, USP14, RNASEL, HDAC1, PLA2G1B, ATL1, VPS4B, UNG, ERMP1, TDG, EXOSC9, USP1, RBBP8, MEST, SEC11A, ATAD1, USP9X, ATAD2, PSMB8, PSMB9, SULF2, FAP, CES1E, XPNPEP3, ATP13A2, PTPN2, LPIN3 |
| BP | GO:0008301 | DNA binding, bending | 7 | 0.01270494 | TOP2A, HHEX, HMGB2, HMGB3, GM21596, HMGB1, CRIP1 |
| BP | GO:0043142 | Single-stranded DNA-dependent ATPase activity | 6 | 0.014472458 | RFC5, RFC3, RAD51, RFC4, RFC2, DSCC1 |
| BP | GO:0004857 | Enzyme inhibitor activity | 10 | 0.0211855 | UGT1A10, PPP1R12A, UGT1A1, CALU, FETUB, TIMP1, HNRNPC, UGT1A9, DYNLL1, DGKZ |
| BP | GO:0042393 | Histone binding | 19 | 0.02162822 | DTX3L, ANP32A, UHRF1, H2AFX, ATAD2, HJURP, SPTY2D1, CKS1B, ATAD2B, CD1D1, A730008H23RIK, NASP, TBL1XR1, CTSL, HAT1, CKS2, NCAPD3, PTMA, MCM2 |
| BP | GO:0051082 | Unfolded protein binding | 14 | 0.021964592 | HSPA9, CCT3, HSP90AA1, PTGES3, HSPE1, PDRG1, DNAJA1, CCT6A, DNAJB1, NUDCD2, DNAJB4, PFDN2, SRSF10, CRYAB |
| BP | GO:0051087 | Chaperone binding | 14 | 0.021964592 | PRNP, TFRC, TIMM9, HSPE1, HSPD1, DNAJA1, STIP1, DNAJB1, BAG5, DNAJB4, DNAJC10, BIRC5, BAX, CTSC |
| BP | GO:0005009 | Insulin-activated receptor activity | 6 | 0.027688811 | MUP19, MUP9, MUP16, MUP1, MUP12, MUP2 |
| BP | GO:0005518 | Collagen binding | 12 | 0.027688811 | SRGN, SPARC, PCOLCE2, LUM, CTSK, PCOLCE, TGFBI, HSD17B12, CTSS, THBS4, CTSB, DDR2 |
| BP | GO:0004197 | Cysteine-type endopeptidase activity | 12 | 0.027688811 | CTSO, CASP8, CTSL, CTSK, USP34, CTSZ, USP1, CTSH, CTSS, CTSC, LGMN, CTSB |
| BP | GO:1990446 | U1 snRNP binding | 5 | 0.029207713 | SNRPD1, SNRPG, SNRPD3, SNRPC, SNRPB |
| BP | GO:0005178 | Integrin binding | 16 | 0.033481411 | ITGB5, ANP32A, THY1, THBS1, THBS4, CTGF, ICAM1, DAB2, COL3A1, FAP, TSPAN8, CD9, TGFBI, PTPN2, FERMT3, FBN1 |
| BP | GO:0032403 | Protein complex binding | 38 | 0.033481411 | ALDH1L1, YWHAB, HDAC1, ARPC1B, PROS1, TWF1, FBLN1, CAPG, HIF1A, RTN4, HSPD1, ICAM1, RAP1B, NRAS, CCNB1, FAM178A, TUBB5, CASP8, CTSL, EPCAM, MYC, CTSH, CTSB, CRTAP, KRT8, MIF, UGT1A6A, UGT1A6B, TGFBR1, REST, CDK7, ABI2, DDX39B, CDK4, PTPRA, BAX, RAN, FBN1 |
| BP | GO:0004175 | Endopeptidase activity | 17 | 0.037290126 | PSMD14, KLK1B24, HTRA3, ADAM10, PSMB8, PSMB9, PSMA3, PSMA4, CASP8, KLK1B4, PSMA1, FAP, KLK1B5, PSMD1, CTSH, ADAMTS9, CTSB |
| CC | GO:0070062 | Extracellular exosome | 338 | 2.9059E-44 | CYFIP2, IFITM3, RPL3, TFRC, NUDT1, NUDT5, CLDN2, PBLD2, ICAM1, LOC100038947, SMC2, C4B, C4A, LGALS3, GJA1, ISLR, NAMPT, STMN1, PSMD1, SNRPD3, B2M, ST6GAL1, SERPINF1, BROX, KRT8, KRT7, MIF, MTAP, TWSG1, PPA1, MXRA8, PSME1, PADI2, KDELR2, AMY1, KCTD12, H2-D1, PLA1A, PFN2, CFH, CFI, VPS26A, THY1, SDSL, MYL12A, MYL12B, PKHD1, PRDX2, CYB5R1, LDHA, TSPAN8, RACGAP1, PRDX1, ZNHIT6, PDGFD, PDGFC, ST3GAL4, APOE, CD14, TSPAN1, STX4A, S100A11, S100A10, PCK2, H2-EB1, CADM1, GP2, CCT6A, BST2, HNRNPM, EHD4, CILP, SDC1, HNRNPC, ITM2A, FERMT3, LGMN, ITM2C, AHCTF1, SLC44A1, ARPC1B, HEXB, CTSZ, HEXA, SERPINA6, AKR1B8, PTPRG, TUBA1B, TUBA1A, NUDCD2, KLK1B4, IMPA1, CTSL, RARS, KLK1B5, CD38, CTSH, QSOX1, SH3BGRL3, TIMP1, TMED7, CTSE, CLIC1, CTSC, CTSB, RAB8B, CCT3, CD53, TMED9, ANXA1, ANXA2, ANXA3, ANXA4, ANXA5, BGN, AKR1A1, SERPINB9, PTGR1, DYNLL1, F3, NME1, RAP2C, UGDH, PLSCR1, PSMA3, TUBB2A, PSMA4, PSMA1, OAF, RHOJ, TFF2, CD48, ALDOC, TAX1BP3, KIFC3, GAS6, CRYAB, PTMA, PAFAH1B2, TMEM106A, CD44, DDR2, C1QB, C1QA, PCNA, AHNAK, RPL12, CAPG, HSPA13, FSTL1, SERPINB1A, NRAS, EXTL2, EPCAM, EXOSC9, SPP1, CKB, GC, CD74, SLC16A1, MDH2, GOLM1, PTGES3, SUSD2, ATAD2, TUBB4B, PSMB8, PSMB9, KRT18, FABP5, AXL, CAPZA1, CLCF1, TAOK1, CAPZA2, PLP2, C1QC, UGT1A10, FAM49B, ALDH1L1, CD84, CD81, CSE1L, PROS1, H2-K1, PEBP1, TM7SF3, CNDP2, ACTG1, FBL, EFEMP2, TUBB5, SLK, BASP1, RUVBL1, CFL1, HIST1H2AE, COTL1, HIST1H2AD, SLC39A5, HIST1H2AG, BANF1, HIST1H2AC, SLC39A4, CAR4, HIST1H2AB, TGM2, SLC30A7, PDIA3, HIST1H2AO, HIST1H2AN, HIST1H2AI, TPM4, HIST1H2AH, SHISA5, ADAM10, PCOLCE, KRT76, PGD, ATP1B1, HIST1H2AP, FCGR3, RBMXL1, SMO, RAB34, S100A6, SRSF2, FETUB, PLAA, SRSF7, DSC2, SNRPB, ATP6V1A, BHLHB9, PSMD14, LYZ2, LYZ1, RHOBTB3, HTRA1, DPT, FBLN1, CORO1A, RTN4, MTM1, RAP1B, DPP7, HNRNPDL, IGFBP7, IGFBP6, RAB6A, HSPA9, PRNP, AMBP, KLK1B24, RARRES2, HPRT, EIF2S1, GGCT, MOB1A, DAB2, PTPRC, PTPRA, CDK1, LCN2, TGFBI, FBN1, SIRPB1B, SIRPB1A, ITGB5, WDR1, COL14A1, CPQ, YWHAB, SNAP23, TRF, LSP1, RND3, SCPEP1, CAPZB, ITGAV, EMILIN1, SKP1A, ACP1, HSP90AA1, MMP7, APAF1, H2AFY, H2AFX, DUSP26, UGT1A6A, CKAP4, UGT1A6B, CDH11, CDHR2, CDH13, PFDN2, SMPDL3A, CRP, COLEC12, ITIH4, USP14, MTPN, RALB, HPGD, PCOLCE2, SEMA3C, VPS4B, H2-Q7, SRSF1, THBS1, THBS4, HSPD1, DNAJB1, CLEC3B, TTR, DNAJB4, STOM, MAL2, HNRNPA1, UGT1A9, SEC11A, MEST, RRM1, DUT, LUM, EGF, HSPE1, DNAJA1, GALE, IMPDH2, XPNPEP3, MGP, CD9, BAX, SSBP1, VIM, PI16, RAN |
| CC | GO:0005634 | Nucleus | 499 | 9.28932E-19 | CYFIP2, ATF2, EHF, RPL3, PGAP2, TFRC, POP4, ELK3, SMC2, ZFP281, PPP4R2, MYC, ZXDC, MYB, NAMPT, PIP4K2A, PSMD1, BBX, RNF111, IER3, DNTTIP2, WDR77, AR, MTAP, DDX39B, WDR82, PRKD3, SARNP, SLBP, SET, CFH, CTBP2, CDCA4, CFI, CDCA8, BCL10, AAAS, HIF1A, NCAPH, PLAC8, LDHA, PRDX1, PDGFC, RRS1, GM21596, S100A11, PLK4, LARP7, CDC7, CDC6, MEX3C, FLI1, HNRNPM, MEX3B, RAD17, HNRNPC, CDK12, PSMG2, HAMP, EZH2, CDKN1C, LIN54, ZFP367, TCF25, NCAPG2, WDR43, MED17, IFIH1, TUBA1A, CSRP2, A730008H23RIK, KLK1B4, TMSB4X, KLK1B5, NUF2, SPIN1, PBK, SH3BGRL3, TRP53INP1, TCF19, SS18, TIGD5, ANXA1, ANXA2, RMI1, TESC, ANXA4, ACTL6A, ANXA5, TCF12, VWA5A, SERPINB9, DYNLL1, GNL3, PLSCR1, PSMA3, TUBB2A, PSMA4, AEBP2, PSMA1, CINP, PLIN2, CRYAB, SGMS1, AHNAK, RNMT, RPL12, SPTY2D1, CENPA, UNG, USP1, ZFP263, RPL13, SRSF10, ATAD1, CENPW, MDH2, STAT1, ATAD2, GTF2H2, TUBB4B, PSMB8, PSMB9, TRMT6, RLIM, NCAPD3, RIF1, CSE1L, HJURP, TUBB5, PAPOLG, RUVBL1, CFL1, KPNA2, HMGN2, HMGN1, SNRPN, UBE2E3, PGD, ILF2, DGKZ, RBMXL1, MAF, MRTO4, HAT1, S100A6, SRSF2, SNRNP27, SRSF3, SNRPF, SNRPC, SRSF7, SNRPB, ZFP330, NOTCH2, RNASEH2A, CBFB, ISG20L2, NREP, GTF2E1, ATXN3, STIP1, DSCR3, BAG5, HNRNPDL, CLCA2, NDN, BRIX1, ZKSCAN1, RBPMS, CNOT6, CDK7, DAB2, CNOT7, CDK4, CDK1, ZFP110, NFE2L2, TRIM30A, MCM7, WDR3, CELF2, CDCA7L, LRMP, METTL14, HHEX, ITGAV, ALYREF2, SKP1A, RFC5, ZFP655, HSP90AA1, RFC3, RFC4, PEG3, DTX3L, CHUK, APAF1, RFC2, ACTR6, DIS3, DUSP26, MUP1, MUP2, TFDP1, MUP9, SNURF, MCM3, BIRC5, MCM4, DNAJC9, MCM5, MCM6, BDP1, DTL, SLC25A4, MCM2, MTPN, 2810417H13RIK, HDAC1, UHRF1, VPS4B, SRSF1, ZBTB43, DNAJB1, DCAF17, RBBP4, RBBP8, SNRPB2, E2F3, HNRNPA1, HNRNPA0, NOP56, DUT, NOP58, PRRX1, MGA, CNBP, DNAJA1, RAD51, IMPDH2, TRIM35, TRMT61A, PTPN2, IFITM3, TCERG1, SPARC, ZAK, NUDT1, PPAN, NUDT5, NUDT4, CDC20, LGALS3, SNRPD1, CHEK1, RPS6KA1, MAGOH, QK, LGALS9, FBXO5, SNRPD3, SKP2, TRIM21, SOX4, TMPO, TIPIN, KRT4, IL1R1, KRT8, KRT7, YOD1, MIF, TIAM1, RRAGA, KCTD10, XRN2, NSMF, BEX4, BEX1, ANP32A, CSTF3, CSTF2, MGST1, TGFA, NMD3, MNDAL, RACGAP1, MIER1, APOE, RBM12, EGR1, CDT1, ZRANB2, BCL11A, GADD45A, INTS2, PARP14, PARP12, ZWINT, MANF, ICK, ANLN, ZZZ3, NOL11, INTS6, TOP2A, AHCTF1, CETN2, BRCA1, PHF11D, ZFP809, SYNCRIP, XPO1, CTSL, REV3L, CD38, CLIC1, APOBEC1, PRMT1, PARP2, ZFR, TSC22D2, PARP9, PALD1, NME1, UGDH, RAD51D, MDFIC, KIF2C, NCOA7, TAX1BP3, PTMA, CD44, VGLL3, DTYMK, PCNA, ZFP704, MIS12, CAPG, TYMS, AIF1, PURB, RANBP3L, SERTAD4, EXOSC9, EXOSC8, LMNA, IL33, RANBP1, MYEF2, NFYA, PTGES3, TMEM176B, ZFP719, RPA1, RPA2, RANBP6, KRT18, RPA3, STK17B, CCNG1, TARDBP, ZFP26, MAD2L1, SPC25, SRP19, DBR1, DSCC1, PRDM5, PEBP1, NUCKS1, TTF2, ACTG1, FBL, RGS2, CCND2, BASP1, COTL1, HIST1H2AE, HIST1H2AD, SNIP1, BANF1, HIST1H2AG, HIST1H2AC, HIST1H2AB, PDIA3, MORC3, HIST1H2AO, HIST1H2AN, KDM2B, LIG1, HIST1H2AI, HIST1H2AH, LMO2, SHISA5, ADAM10, CYBA, KRT76, UTP11L, HIST1H2AP, BRMS1, LOX, PSPC1, TBL1XR1, PLAA, GEMIN6, PPID, BHLHB9, PSMD14, RPS19BP1, ZCCHC9, HTRA2, TOR1AIP2, NUP160, CCNB2, SYCE2, CCNB1, LLPH, ECT2, HSPA9, UBE2I, AUTS2, UBE2C, CAV2, CBX3, ALYREF, KLK1B24, NFXL1, EIF2S1, NMRAL1, NASP, UBE2S, UBA2, SAE1, PICALM, KDM5A, YWHAB, CHD9, HMGB2, HMGB3, HMGB1, AFF3, SENP7, FAM178A, CASP8, TOPBP1, RBM17, TTC37, PTGIS, H2AFY, NCBP2, MMP2, H2AFX, MUP16, DDX50, MUP12, DCK, CCNA2, SUPV3L1, ELF1, RBL1, MUP19, PALLD, IRF8, PFDN2, NUP54, SKIV2L2, FAM60A, TAF9, POMP, LIN9, ATAD2B, PABPN1, FAM120C, TDG, TRA2B, POLD2, TRA2A, BUB3, NIPSNAP3B, RRM2, TAF15, NIFK, IFI204, IFI205, HN1, NAP1L1, SSRP1, NAP1L4, REST, POLE2, ALOX5AP, RGS10, BAX, FAM98B, SSBP1, LPIN3, RAN |
| CC | GO:0005615 | Extracellular space | 162 | 4.07968E-12 | SPARC, TFRC, SERPINE2, SCT, 1810009J06RIK, PROS1, NUDT1, PEBP1, LOXL1, ICAM1, CTGF, ACTG1, C4B, C4A, LGALS3, BMPER, CFL1, CHEK1, NAMPT, DPYSL3, B2M, PDIA3, POSTN, IL1R1, IGFBP4, SERPINF1, WNT5A, PCOLCE, FRMD4B, UTP11L, MIF, TWSG1, RBMXL1, LOX, MTHFD2, PLBD1, FETUB, AMY1, HAMP2, CFH, LYZ2, CFI, LYZ1, TGFA, DPT, FBLN1, PLA2G7, PRDX1, PDGFD, PDGFC, CLCA1, GM21596, IGFBP7, APOE, IGFBP6, CD14, STX4A, S100A11, CRTAP, AMBP, KLK1B24, LY86, MANF, CILP, KITL, SERPINB6B, LCN2, TGFBI, HAMP, FBN1, COL14A1, CPQ, HEXB, CTSZ, HMGB2, TRF, HMGB1, SERPINA6, CTSS, PTPRG, ADAMTS5, CTSO, FAM178A, KLK1B4, CTSL, CES2G, CTSK, KLK1B5, CTSH, QSOX1, CPXM1, TIMP1, GM2663, ADAMTS9, CLIC1, HGFAC, CTSC, CTSB, SRGN, PTGIS, ANXA1, MMP7, ANXA2, GIF, MMP2, SFTPD, ANXA5, AKR1A1, MUP16, SERPINB9, MUP12, MUP1, F3, 1190002N15RIK, MUP2, GNL3, GAL, MUP19, MUP9, CDH13, TFF2, DNAJC9, LTB, GAS6, CRELD2, SMPDL3A, CRP, SEMA3C, PLA2G1B, PTN, THBS1, FSTL1, THBS4, HSPD1, SERPINB1A, CCL9, CLEC3B, CCL8, TTR, CCL6, SPP1, AGR2, STOM, GC, CBR3, IL33, CMTM3, EGF, LUM, GOLM1, SULF2, COL3A1, FAP, CES1E, AXL, CLCF1, MGP, CD9, PI16, CKLF, PF4, C1QC |
| CC | GO:0005654 | Nucleoplasm | 189 | 1.49139E-10 | ATF2, EHF, PID1, RPL3, CSE1L, DSCC1, CNDP2, ELK3, CDC20, FBL, CCND2, PPP4R2, ZFP281, PAPOLG, MYC, CHEK1, NAMPT, RUVBL1, MYB, BBX, SNIP1, BANF1, SNRPD3, FBXO5, KPNA2, RNF111, SOX4, MORC3, TIPIN, LIG1, COG3, KRT8, MRPS18B, MIF, ILF2, DGKZ, WDR77, DDX39B, PRKD3, PSPC1, TBL1XR1, KCTD10, SARNP, MRTO4, HAT1, RBP1, NSMF, GEMIN6, SRSF7, PPID, NOTCH2, NUP205, SET, RNASEH2A, RPS19BP1, TUBGCP2, CSTF3, CSTF2, ISG20L2, NMD3, AAAS, SYCE2, RACGAP1, HNRNPDL, CLEC7A, MIER1, NDN, RBM12, ARGLU1, GRIA3, EGR1, ZRANB2, UBE2I, RBPMS, CBX3, GADD45A, BCL11A, LARP7, CDC7, CDC6, ANLN, HNRNPM, CNOT7, NASP, CDK4, CDK1, UBA2, RAD17, HNRNPC, INTS6, EZH2, TOP2A, AHCTF1, ZFP367, MCM7, SLC44A1, SNAP23, BRCA1, AFF3, MED17, SYNCRIP, METTL14, CASP8, RARS, PMEPA1, TTC37, LRRC45, HSP90AA1, ANXA1, DTX3L, CHUK, PEG3, PARP2, PRMT1, DIS3, NCBP2, ACTL6A, H2AFX, ZFR, VWA5A, HAUS3, PARP9, GNL3, CCNA2, UGDH, ELF1, RAD51D, RBL1, PSMA1, MDFIC, MCM3, IRF8, MCM4, MCM5, PFDN2, MCM6, BDP1, PTMA, DTL, DLD, SKIV2L2, MCM2, PCNA, HPGD, RNMT, HDAC1, UHRF1, SRSF1, POMP, CAPG, LIN9, ADD3, BTAF1, RBBP4, TDG, DNAJB4, EXOSC9, LMNA, POLD2, ZFP263, RBBP8, SNRPB2, E2F3, BUB3, HNRNPA1, SRSF10, CBR3, HNRNPA0, NOP56, DUT, TAF15, MYEF2, IFI204, NIFK, NFYA, MDH2, STAT1, PTGES3, MGA, ATAD2, RPA1, RPA2, RAD51, POLE2, RPA3, RLIM, NUP35, TARDBP, RAN |
| CC | GO:0031012 | Extracellular matrix | 50 | 3.50365E-09 | SERPINE2, COL14A1, LOXL1, ACTG1, LGALS3, ADAMTS5, ADAMTS2, TUBB5, CFL1, EMILIN1, TIMP1, TGM2, SBSPON, POSTN, HSP90AA1, MMP7, SERPINF1, MMP2, BGN, PCOLCE, F3, CKAP4, PLSCR1, MATN2, FBN2, RPL12, RPN1, HTRA1, DPT, FBLN1, THBS1, THBS4, HSPD1, PRDX1, LMNA, IGFBP7, APOE, HSPA9, LUM, RARRES2, TUBB4B, CCT6A, HNRNPM, COL3A1, CILP, MGP, TGFBI, VIM, RAN, FBN1 |
| CC | GO:0009986 | Cell surface | 81 | 4.17389E-09 | IFITM3, SPARC, TFRC, ITGB5, H2-K1, TRF, PEBP1, CTSS, ICAM1, LGALS3, CD1D1, MRC1, SLC39A6, CD38, ITGAV, CCR5, ADAMTS9, CD34, CAR4, CDON, CTSB, CD53, PDIA3, HSP90AA1, FCER1G, ANXA1, MMP7, ANXA2, KRT4, SCARA5, IL1R1, ANXA4, WNT5A, ATPIF1, F2R, BGN, ADAM10, MIF, F3, CKAP4, TGFBR1, PDIA4, TGFBR2, TYROBP, MXRA8, CRYAB, CD44, H2-D1, NOTCH2, USP14, IGSF5, PLA2G1B, H2-Q9, SDC2, H2-Q7, TGFA, PTN, THY1, THBS1, HSPD1, EPCAM, PDGFC, APOE, CD14, STX4A, ABCA1, CD74, PRNP, AMBP, CAV2, DCBLD2, SULF2, BST2, HNRNPM, PTPRC, FAP, AXL, SDC1, CD9, FOLR2, H2-AB1 |
| CC | GO:0005737 | Cytoplasm | 492 | 4.23487E-09 | CYFIP2, PNMT, ATF2, PID1, RPL3, TFRC, ELK3, SMC2, GJA1, PPP4R2, MYC, PPP4R4, STMN1, NAMPT, DPYSL3, PIP4K2A, BBX, RNF111, TSTD1, QTRTD1, WDR77, AR, MTAP, DDX39B, PRKD3, RBP1, PSME1, PADI2, SLBP, NUP205, SET, CFH, CDCA3, CDCA8, VPS26A, SLAIN2, BCL10, AAAS, PLA2G7, HIF1A, NCAPH, ANAPC10, MTMR7, PRDX2, LDHA, RNF213, PRDX1, PDGFC, GM21596, S100A11, PLK4, LARP7, CDC7, CDC6, MEX3C, MEX3B, KITL, PLCH1, EZH2, CDKN1C, BTG3, ARPC1B, IFIH1, TUBA1B, TUBA1A, A730008H23RIK, IMPA1, RARS, TMSB4X, SPIN1, SH3BGRL3, 2610301B20RIK, CEP55, TRP53INP1, 2700097O09RIK, LRRC45, ANXA1, ANXA2, ANXA3, TESC, ANXA4, ANXA5, TCF12, BGN, SERPINB9, DYNLL1, PTGR1, F3, PSMA3, TUBB2A, PSMA4, PSMA1, PLIN2, KIFC3, GAS6, CRYAB, PAFAH1B2, RNASEL, OAT, AHNAK, ATL1, RPL12, SERPINB1A, 4932431P20RIK, SPP1, ZFP263, CKB, PAIP1, STAT1, USP9X, PBDC1, TUBB4B, PSMB8, GNMT, PSMB9, FABP5, ABI2, TAOK1, RLIM, ALDH1L1, NCF1, RIF1, CSE1L, HJURP, F13A1, SLK, TUBB5, 1700017B05RIK, RNF19A, PAPOLG, RUVBL1, CFL1, PLS3, KPNA2, HMGN2, TMSB10, HMGN1, SNRPN, TPM4, UBE2E3, TPM1, FRMD4B, PGD, ILF2, DGKZ, MAF, SMO, STIM1, RAB34, MRTO4, S100A6, SRSF3, SNRPF, PHLDB2, SRSF7, SNRPB, TRIM34A, NOTCH2, RNASEH2A, KCNE3, FBLIM1, TUBGCP2, LYZ2, LYZ1, RHOBTB3, NREP, CORO1A, GTF2E1, AK7, MTM1, RAP1B, ATXN3, STIP1, HNRNPDL, NDN, IGFBP6, EIF2B3, RBPMS, HPRT, CNOT6, CDK7, DAB2, CNOT7, SERPINB6B, CDK4, CDK1, ZFP110, NFE2L2, TRIM30A, MCM7, WDR1, CPQ, CELF2, CDCA7L, SNAP23, APIP, LRMP, HHEX, CAPZB, ALYREF2, SKP1A, ACP1, HGFAC, CCDC50, ZFP655, HSP90AA1, PPP1R12A, PEG3, DTX3L, CHUK, APAF1, SGIP1, ACTR6, DIS3, HAUS4, HAUS3, DUSP26, HAUS1, MCM3, BIRC5, DNAJC9, BDP1, DTL, MCM2, ITIH4, MTPN, 2810417H13RIK, HDAC1, VPS4B, SRSF1, EHBP1, DNAJB1, CLEC3B, DNAJB4, EIF4H, E2F3, HNRNPA1, NOP56, DUT, NOP58, MGA, CNBP, ATOX1, BICC1, DNAJA1, RAD51, IMPDH2, TRIM35, PTPN2, IFITM3, SPARC, ARL6IP1, ZAK, LST1, NUDT1, PBLD2, NUDT4, CDC20, LGALS3, SNRPD1, CHEK1, RPS6KA1, MAGOH, QK, LGALS9, FBXO5, SNRPD3, SKP2, TRIM21, B2M, SOX4, TIPIN, COG3, ARRDC3, KRT8, KRT7, MIF, TIAM1, RRAGA, PPA1, KCTD10, NSMF, RIN2, MMADHC, PFN2, BEX4, BEX1, ANP32A, TWF1, NMD3, MNDAL, PKHD1, SNX4, RACGAP1, INPP5J, DYM, APOE, TSPAN1, STX4A, SNX5, TMEM184A, EGR1, BCL11A, GADD45A, PARP14, ZWINT, CCT6A, ICK, BST2, ANLN, SDC1, AHCTF1, CETN2, BRCA1, KRT20, AKR1B8, SYNCRIP, XPO1, NUDCD2, CTSL, CTSK, CCR5, BCL2A1A, CD34, CLIC1, CTSB, CCT3, APOBEC1, PARP3, PRMT1, PARP2, ZFR, TSC22D2, SFTPD, PARP9, ZDHHC17, PALD1, CTTNBP2NL, NME1, PDRG1, RAP2C, RAD51D, GAL, MDFIC, 2310022B05RIK, KIF2C, ALDOC, TAX1BP3, AIDA, MAPRE1, PTMA, CD44, PCNA, CAPG, PTN, TYMS, AIF1, CRIP1, PTS, EXOSC9, EXOSC8, LMNA, UBTD2, CBR3, MAP4K4, RANBP1, MYEF2, PTGES3, TUBE1, RANBP6, PLEKHA8, TTC8, KRT18, GSTA4, CAPZA1, TARDBP, MAD2L1, SRP19, ZFAND5, PEBP1, NUCKS1, TTF2, LOXL1, ACTG1, CNDP2, RGS5, RGS2, CCND2, PPIP5K2, BASP1, COTL1, SNIP1, BANF1, TGM2, PDIA3, SLC30A7, POSTN, WNT5A, ADAM10, CYBB, CYBA, UTP11L, KLHL42, BRMS1, FAM161A, PSPC1, PLAA, GEMIN6, PPID, BHLHB9, RPS19BP1, COPB1, HTRA1, KLHL11, DTX4, RTN4, CCNB1, ECT2, HSPA9, PRNP, PNPT1, UBE2I, UBE2C, CAV2, ALYREF, DENND4C, EIF2S1, NMRAL1, NASP, UBE2S, ATG16L1, SRPRB, UBA2, LSM14A, RTP4, KDM5A, YWHAB, CHD9, PLEK, HMGB2, HMGB3, HMGB1, AFF3, CD1D1, CASP8, BLNK, TOPBP1, TTC37, PTGIS, NCBP2, MMP2, SPATS2, ARAP2, CKAP4, TGFBR2, CCNA2, SUPV3L1, PSRC1, RASA3, PALLD, CDH11, CDH13, NAA35, PFDN2, RBMS3, USP14, RGS19, HPGD, POMP, ADD3, THBS1, HSPD1, PABPN1, STOM, BNIP2, NIPSNAP3B, RRM1, RRM2, TAF15, NIFK, CMTM3, IFI204, IFI205, NAP1L1, SSRP1, HSPE1, FMNL2, RGS10, BAX, FAM98B, VIM, RAN |
| CC | GO:0005576 | Extracellular region | 161 | 4.79238E-07 | SPARC, TFRC, SERPINE2, SCT, PROS1, F13A1, LOXL1, CTGF, C4B, C4A, LGALS3, ISLR, EFEMP2, BMPER, CFL1, NAMPT, LGALS9, PAMR1, B2M, PDIA3, POSTN, ST6GAL1, IL1R1, IGFBP4, SERPINF1, WNT5A, PCOLCE, MIF, TWSG1, CCDC80, LOX, FETUB, AMY1, NBL1, EPHA3, PLA1A, FBN2, NOTCH2, HAMP2, CFH, LYZ2, HTRA3, CFI, LYZ1, HTRA1, TGFA, DPT, FBLN1, PLA2G7, DPP7, PDGFD, PDGFC, PENK, CLCA1, GM21596, IGFBP7, WFDC18, APOE, WFDC17, IGFBP6, CD14, CRTAP, CTLA2A, AMBP, RARRES2, GP2, LY86, MANF, CILP, KITL, CALU, LCN2, SDC1, TGFBI, HAMP, FBN1, COL14A1, CPQ, HMGB2, TRF, TREM2, HMGB1, SERPINA6, SCPEP1, ADAMTS5, ADAMTS2, GLIPR1, QSOX1, EMILIN1, CPXM1, TIMP1, CD34, HGFAC, CTSB, SBSPON, SRGN, ANXA1, MUP10, MMP7, ANXA2, GIF, MMP2, TNFRSF19, SFTPD, MUP15, BGN, MUP16, MUP7, MUP12, MUP1, 1190002N15RIK, MUP13, MUP2, MUP14, GAL, MUP8, MUP19, MUP9, TFF2, GAS6, CRELD2, SMPDL3A, MATN2, CRP, ITIH5, C1QB, ITIH4, C1QA, SEMA3C, PCOLCE2, PLA2G1B, CAPG, PTN, THBS1, FSTL1, THBS4, CCL9, CLEC3B, CCL8, TTR, CHSY1, CNPY4, CCL6, SPP1, AGR2, GC, IL33, GM2083, EGF, LUM, LGI2, MFAP5, COL3A1, FAP, CLCF1, MGP, CD9, PI16, CKLF, PF4, C1QC |
| CC | GO:0005730 | Nucleolus | 90 | 2.64261E-06 | TOP2A, KDM5A, TCERG1, SRP19, RPL3, RPS4L, WDR3, CDCA7L, HMGB2, PPAN, HJURP, WDR43, SMC2, FBL, RGS2, XPO1, CCND2, A730008H23RIK, CTSL, MYC, REV3L, SPIN1, SKP2, RNF111, TRP53INP1, CTSB, ZFP655, H2AFY, KDM2B, PARP2, DIS3, DNTTIP2, UTP11L, DDX50, ILF2, GNL3, PLSCR1, MDFIC, PSPC1, MRTO4, XRN2, PFDN2, DTL, SKIV2L2, PPID, PAFAH1B2, ZFP330, RPS19BP1, RNMT, RPL12, ZCCHC9, ISG20L2, CDCA8, CAPG, NMD3, TYMS, SPTY2D1, MNDAL, DNAJB1, DCAF17, RNF213, PRDX1, EXOSC9, EXOSC8, TRA2A, RRS1, RBBP8, SNRPB2, BRIX1, LLPH, PLK4, HSPA9, NOP56, NOP58, IFI204, NIFK, PRRX1, IFI205, STAT1, HN1, SSRP1, DAB2, ZZZ3, KRT18, RAD51, CDK4, RAD17, CDK12, NOL11, RAN |
| CC | GO:0005829 | Cytosol | 155 | 2.77784E-05 | NCF1, ARL6IP1, SERPINE2, CSE1L, CNDP2, CTGF, ACTG1, GJA1, RGS2, TUBB5, CCND2, PPIP5K2, SNRPD1, CFL1, STMN1, NAMPT, MYB, DPYSL3, DENND5A, SNRPD3, KPNA2, TGM2, YOD1, MAT1A, WDR77, TIAM1, RBP1, SNRPG, S100A6, GEMIN6, SNRPF, SNRPB, PFN2, ATP6V1A, NOTCH2, SET, CDCA3, ANP32A, RHOBTB3, HTRA1, HTRA2, VPS26A, BCL10, THY1, HIF1A, CORO1A, AK7, GSPT1, MTMR7, RAP1B, ATXN3, STIP1, PRDX2, LDHA, DPP7, BAG5, RNF213, PRDX1, PDGFC, NDN, APOE, RAB6A, SNX5, UBE2I, CAV2, DENND4C, HPRT, GGCT, ICK, CDK7, CDK4, ATG16L1, LCN2, UBA2, PLCH1, HNRNPC, PSMG2, SAE1, NFE2L2, ZFP110, MCM7, YWHAB, SCPEP1, TUBA1B, CASP8, TMSB4X, PGM3, CTSH, SKP1A, TRP53INP1, HSP90AA1, ANXA1, APAF1, ANXA2, CHUK, PRMT1, ANXA5, AKR1A1, MUP16, SERPINB9, DYNLL1, PARP9, MUP12, DCK, MUP1, MUP2, PALD1, TGFBR2, NME1, RAP2C, UGDH, PLSCR1, PSMA3, PSMA4, PSRC1, PSMA1, MUP19, MUP9, PTRH2, BIRC5, PLIN2, PFDN2, RAPGEF6, CRYAB, PAFAH1B2, MTPN, DTYMK, AHNAK, HDAC1, VPS4B, POMP, AIF1, HSPD1, DNAJB1, RANBP3L, DNAJB4, BNIP2, GC, SRSF10, CBR3, NIPSNAP3B, DUT, STAT1, CNBP, PSMB8, ATOX1, PSMB9, DNAJA1, REST, ABI2, ALOX5AP, RGS10, BAX, VIM, MAD2L1 |
| CC | GO:0034709 | Methylosome | 8 | 7.77018E-05 | SNRPD1, PRMT1, SNRPG, ERH, SNRPD3, SNRPF, WDR77, SNRPB |
| CC | GO:0005694 | Chromosome | 44 | 0.000111552 | AHCTF1, RIF1, ZWILCH, MIS12, HMGB2, HJURP, CDCA8, HMGB3, LRMP, BRCA1, HMGB1, CENPA, NCAPH, SMC2, FBL, SYCE2, SMCHD1, NUDCD2, NUF2, RBBP8, BANF1, GM21596, BUB3, TOPBP1, LLPH, TMPO, IL33, CENPW, H2AFY, NIFK, HIST1H2AH, H2AFX, ZFR, SSRP1, ZWINT, RAD51D, CFDP1, BIRC5, KIF2C, DTL, SPC25, ZFP330, MAD2L1, EZH2 |
| CC | GO:0005687 | U4 snRNP | 7 | 0.000316626 | SNRPN, DDX39B, SNRPD1, SNRPG, SNRPD3, SNRPF, SNRPB |
| CC | GO:0048471 | Perinuclear region of cytoplasm | 70 | 0.00044244 | ANKRD13C, CYFIP2, TFRC, YWHAB, HMGB2, TRF, CTGF, CDC20, SLK, MYC, STBD1, LAMP3, CLIC1, CD34, RAB8B, CAR4, CTSB, SLC30A7, HSP90AA1, ANXA2, SERPINF1, ANXA4, CYBB, CKAP4, NME1, RAB34, MCM3, S100A6, CDH13, MTPN, 2810417H13RIK, SEC23A, SET, ANP32A, HDAC1, TWF1, TGFA, BCL10, PTN, AIF1, PKHD1, BAG5, TRA2B, LMNA, SPP1, EIF4H, STOM, BNIP2, GC, STX4A, TSPAN1, TMEM184A, ABCA1, CAV2, STAT1, PTGES3, NMRAL1, MANF, DNAJA1, CDK7, DAB2, RAD51, EHD4, CDK4, CCNG1, BAX, VIM, PICALM, ITM2C, MAD2L1 |
| CC | GO:0016363 | Nuclear matrix | 19 | 0.000471053 | TCERG1, MORC3, RNASEL, AHCTF1, SPARC, ANP32A, UHRF1, KRT8, MAT1A, HNRNPM, KRT18, DDX39B, PSPC1, LMNA, HAT1, CFL1, RUVBL1, NDN, NSMF |
| CC | GO:0000785 | Chromatin | 22 | 0.000488962 | AHCTF1, H2AFY, CBX3, HDAC1, DSCC1, H2AFX, HTRA2, RPA2, NUCKS1, REST, FAM178A, RAD51, CCND2, CDK4, WDR82, CHEK1, HMGN2, RAN, MCM2, NFE2L2, HMGN1, TMPO |
| CC | GO:0005783 | Endoplasmic reticulum | 115 | 0.000606285 | ANKRD13C, IFITM3, UGT1A10, PGAP2, ARL6IP1, H2-K1, GJA1, ADORA1, SLC39A6, TGM2, PDIA3, SCARA3, ST6GAL1, FNDC3B, SHISA5, CYBB, PDIA5, PDIA4, RAB32, CYP39A1, STIM1, SOAT1, MAL, FKBP7, KDELR2, H2-D1, SET, ANP32A, RPN1, MGST1, ILDR2, TOR1AIP2, THY1, TMED11, UGT1A7C, RTN4, RRS1, SSR1, APOE, S100A10, SEC16B, NUS1, PRNP, CRTAP, CAV2, EPHX1, MANF, EXT1, EHD4, ARMC10, DNAJC10, CALU, STT3A, SRPRB, CNIH4, SLC26A6, FKBP14, CPQ, CCDC47, CTSZ, LRMP, SYNCRIP, JPH3, STBD1, DSE, QSOX1, TMED7, CTSC, VKORC1, MESDC2, TMED9, PTGIS, UGT1A1, SSR2, ANXA5, UGT1A6A, CKAP4, DDOST, UGT1A6B, RCN1, PIGA, TRIM59, CRELD2, SEC23A, SGMS1, ATL1, H2-Q7, POMP, HSD17B12, PTN, THBS1, HSPA13, THBS4, EXTL2, ERMP1, KDELC1, UGT1A5, AGR2, STOM, UGT1A2, UGT1A9, MEST, SEC11A, CD74, CNBP, MBOAT1, FMO1, SULF2, DNAJA1, CES1E, MGP, ALOX5AP, BAX, ESYT2, PTPN2 |
| CC | GO:0043231 | Intracellular membrane-bounded organelle | 72 | 0.001481345 | TFRC, CTSZ, TREM2, CTSS, CTGF, ELK3, SYNCRIP, FAM178A, XPO1, RUVBL1, CHEK1, AP1S2, CD38, PMEPA1, QSOX1, AASS, CTSB, VKORC1, TIPIN, CHUK, UGT1A1, LIG1, ADAM10, PGD, GTPBP2, ZDHHC17, UGT1A6A, DDOST, UGT1A6B, CYP39A1, RRAGA, PSMA4, SMO, SARNP, HAT1, MCM3, PLIN2, DTL, UGT8A, COPB1, POMP, MGST1, VPS26A, HSPA13, UGT1A7C, HSPD1, BTAF1, DPP7, TRA2A, BNIP2, UGT1A2, UGT1A9, SEC11A, ABCA1, SEC16B, AMBP, PTGES3, EPHX1, FMO1, ZWINT, DNAJA1, ANLN, DAB2, ARHGAP30, PTPRA, POLE2, CALU, NUP35, ATP13A2, PLCH1, LSM14A, SLC26A6 |
| CC | GO:0042555 | MCM complex | 6 | 0.002242468 | MCM7, MCM3, MCM4, MCM5, MCM6, MCM2 |
| CC | GO:0016324 | Apical plasma membrane | 38 | 0.002850951 | ATP6V1A, IGSF5, CD81, TRF, THY1, PKHD1, GJA1, P2RY6, EPCAM, SLCO2B1, PDPN, MAL2, CD34, STX4A, SLC39A4, CTSB, CAR4, PDIA3, HSP90AA1, ANXA1, GIF, ANXA4, AKR1A1, GP2, CYBA, ATP1B1, TGFBR1, BST2, CLDN4, DAB2, CDHR2, MAL, BIRC5, CD9, RAPGEF6, CD44, SLC26A6, DDR2 |
| CC | GO:0005578 | Proteinaceous extracellular matrix | 37 | 0.002850951 | FBN2, SPARC, COL14A1, DPT, FBLN1, HSD17B12, PTN, THBS4, LOXL1, CTGF, LGALS3, ADAMTS5, ADAMTS2, CLEC3B, EFEMP2, EMILIN1, TIMP1, ADAMTS9, TGM2, SBSPON, POSTN, CRTAP, MMP7, ANXA2, LUM, MMP2, WNT5A, SFTPD, BGN, MFAP5, COL3A1, CCDC80, LOX, CILP, TGFBI, MATN2, FBN1 |
| CC | GO:0005913 | Cell-cell adherens junction | 37 | 0.002850951 | AHNAK, YWHAB, TWF1, CAPG, RTN4, DNAJB1, CCNB2, LDHA, SLK, CAPZB, RARS, PRDX1, RUVBL1, EFHD2, EIF4H, CLIC1, ARGLU1, SNX5, TMPO, NOP56, RANBP1, ANXA1, CADM1, ANXA2, STAT1, ANLN, KRT18, FMNL2, EHD4, ABI2, CAPZA1, ESYT2, MAPRE1, PHLDB2, DSC2, RAN, PICALM |
| CC | GO:0009897 | External side of plasma membrane | 37 | 0.002911157 | CD83, TFRC, H2-Q9, H2-M3, H2-K1, THY1, THBS1, ICAM1, AMOT, LGALS3, TMEM123, CD1D1, CTSL, PDPN, ITGAV, CD14, CCR5, CD34, B2M, CTSB, ABCA1, CD74, FCER1G, ANXA5, H2-AA, TGFBR2, FCGR1, FCGR3, PTPRC, CDH13, SDC1, CD9, CD48, LY6A, CD44, H2-D1, H2-AB1 |
| CC | GO:0043234 | Protein complex | 61 | 0.003190314 | TOP2A, ALDH1L1, YWHAB, PROS1, HMGB2, BRCA1, CDC20, TUBB5, CASP8, KLK1B4, MYC, RUVBL1, KLK1B5, RNF111, RBM17, HSP90AA1, ANXA1, ANXA2, IL1R1, PRMT1, ACTL6A, LMO2, TPM1, UGT1A6A, DDOST, TGFBR1, UGT1A6B, AR, STIM1, DDX39B, HAT1, NUP54, CD48, CD44, SET, CBFB, HDAC1, CDCA8, BCL10, CORO1A, MYL12A, RTN4, HSPD1, MYL12B, STIP1, SNX4, PRPSAP2, TTR, RBBP4, GRIA3, CAV2, PTGES3, KLK1B24, REST, NASP, CDK4, MGP, SDC1, HNRNPC, RAN, PF4 |
| CC | GO:0000776 | Kinetochore | 19 | 0.005120257 | AHCTF1, CENPW, PPP1R12A, ZWILCH, MIS12, DYNLL1, CENPA, WDR43, NUP160, ZWINT, NUDCD2, XPO1, CFDP1, NUF2, BIRC5, KIF2C, BUB3, SPC25, MAD2L1 |
| CC | GO:0031982 | Vesicle | 23 | 0.005483284 | SLC30A7, SPARC, ANXA1, KCNE3, ANXA2, AHNAK, CD81, TRF, VPS26A, MIF, DPP7, BASP1, RAB34, LAMP3, CFL1, SPP1, STOM, ATP13A2, TSPAN1, CLIC1, SLC26A6, PF4, PICALM |
| CC | GO:0000793 | Condensed chromosome | 9 | 0.00554558 | TOP2A, RAD51, H2AFY, RPA1, HMGB2, BANF1, NCAPD3, BRCA1, SMC2 |
| CC | GO:0016020 | Membrane | 466 | 0.006012893 | CYFIP2, ATF2, PGAP2, SERPINE2, TFRC, H2-L, CLDN2, GJA1, STMN1, AP1S2, C3AR1, PIP4K2A, PSMD1, IER3, FNDC3B, QTRTD1, CLDN4, PRKD3, CLDN9, H2-D1, NUP205, TTYH1, CFI, RPN1, VPS26A, THY1, BCL10, AAAS, NCAPH, MTMR7, CYB5R1, LDHA, RNF213, PDGFD, PDGFC, ST3GAL4, SSR1, ABCA1, B3GALNT1, CADM1, ABCA9, GP2, HNRNPM, EHD4, KITL, FXYD1, SSPN, PLCH1, HNRNPC, FXYD5, FERMT3, HEXB, FCRLS, NCAPG2, HEXA, MS4A4D, OSBPL11, MED17, JPH3, RARS, MRC1, NUF2, CEP55, ANXA1, ANXA2, GPR34, ANXA3, TESC, SSR2, ANXA5, EMP1, LRRC40, SLC39A10, SERPINB9, EMP3, TIMM21, DYNLL1, F3, GNL3, MS4A6B, FAM210B, PLSCR1, PIGA, PTRH2, GAS1, PLIN2, KIFC3, TMEM106A, SGMS1, AHNAK, ATL1, TIMM9, RPL12, ADCY7, MS4A6D, MS4A6C, SERPINB1A, NRAS, EXTL2, ERMP1, EPCAM, GCNT2, RPL13, ATAD1, SLC16A1, MDH2, USP9X, GOLM1, LAPTM5, SNX24, FMO1, DCBLD2, RNF145, 9530068E07RIK, NCAPD3, ANKRD13C, UGT1A10, FAM49B, NCF1, CSE1L, H2-K1, CASC4, TM7SF3, IYD, FCGRT, SLCO2B1, LAMP3, RNF19A, PAPOLG, CFL1, ADORA1, SLC39A6, SLC39A5, KPNA2, SLC39A4, IL13RA1, TPM4, F2R, FAM118A, CASD1, ILF2, DGKZ, RAB32, FCGR1, TPST1, ACAP2, FCGR3, RAB31, RBMXL1, ARMCX2, SMO, STIM1, RAB34, S100A6, MAL, ERH, LY6A, PHLDB2, DSC2, NOTCH2, KCNE3, CBFB, TUBGCP2, H2-M3, ILDR2, CORO1A, MTM1, RAP1B, BAG5, CLCA2, RAB6A, GRIA3, SLC38A5, SLC38A4, SEC16B, CNOT6, CDK7, DAB2, MFSD7B, CNOT7, PTPRC, CDK4, PTPRA, SYCN, CDK1, CNIH4, SLC26A6, H2-AB1, ST6GALNAC2, ITGB5, MCM7, CCDC47, SNAP23, TRF, TREM2, LRMP, LSP1, CAPZB, DSE, EFHD2, PMEPA1, ITGAV, HSP90AA1, UGT1A1, SGIP1, DIS3, TNFRSF19, UGT1A6A, DDOST, ADORA2A, MCM3, MCM4, DNAJC9, TRIM59, MCM5, DTL, SLC25A4, SEC23A, VPS4B, H2-Q7, HSD17B12, EHBP1, AP3M1, P2RY6, DCAF17, DNAJB4, EIF4H, MAL2, UGT1A2, HNRNPA1, UGT1A9, SEC11A, MEST, SLC25A24, NOP56, SLC35A1, SLC35A4, NOP58, MBOAT1, TM6SF1, DNAJA1, IMPDH2, SLC25A30, PI16, FOLR2, PTPN2, SLC25A35, IFITM3, IFITM2, PLEKHB2, ARL6IP1, LST1, NUDT1, ICAM1, LGALS3, B2M, TMPO, CDON, ST6GAL1, COG6, IL1R1, BROX, SLC35C1, COG3, ARRDC3, TM4SF4, CYP39A1, TIAM1, MARF1, SOAT1, XRN2, MXRA8, KDELR2, NSMF, KCTD12, EPHA3, IGSF5, UGT8A, SDC2, MGST1, TGFA, NMD3, UGT1A7C, MNDAL, SNX4, TSPAN8, RACGAP1, TSPAN7, DYM, CD14, APOE, TSPAN1, STX4A, GPM6B, SNX5, TMEM184A, NUS1, H2-EB1, TMEM184C, INTS2, IRGM2, PARP14, EXT1, BST2, ARMC10, CALU, SDC1, ITM2A, ITM2C, SLC44A1, AP4E1, CTSS, EDA2R, PTPRG, SYNCRIP, GLIPR1, XPO1, STBD1, CD38, QSOX1, CCR5, CD34, CLIC1, CTSC, RAB8B, CD53, VKORC1, TMED9, FCER1G, PRMT1, PARP9, ZDHHC17, NME1, RAP2C, RHOJ, KIF2C, CD48, TAX1BP3, RHOV, AIDA, CD44, DDR2, TOR4A, ZDHHC20, PRIM1, PTN, TYMS, AIF1, PDPN, MPZL2, CD74, SUSD2, TMEM176B, TMEM176A, PLEKHA8, DPY19L1, TTC8, FAP, AXL, CAPZA1, STK17B, CAPZA2, ESYT2, CD68, CD84, CD83, CD81, TMEM140, ACTG1, RGS5, FBL, RGS2, CCND2, BASP1, FAM198B, CAR9, DENND5A, SBF2, CAR4, TGM2, SLC30A7, SCARA3, SCARA5, SHISA5, ADAM10, CYBB, CYBA, ATP1B1, ATAD3A, MPEG1, TYROBP, TMX2, APLNR, COX18, COPB1, SLC41A3, HTRA1, HTRA2, TOR1AIP2, TMED11, RTN4, CCNB2, TMEM123, CCNB1, PRNP, PNPT1, CAV2, LHFP, DENND4C, EPHX1, NFXL1, EIF2S1, ATG16L1, OSTC, DNAJC10, STT3A, SRPRB, PICALM, RTP4, YWHAB, PLEK, SLC40A1, RND2, RND3, CD1D1, BLNK, ABCC4, PTGIS, MMP2, DDX50, CKAP4, TGFBR1, TGFBR2, IGDCC4, CDH11, CDHR2, CDH13, NUP54, LTB, B4GALT6, SCN4B, TMEM45A, COLEC12, USP14, RGS19, RALB, POMP, ADD3, HSPD1, CYTH4, CHSY1, STOM, CMTM3, IFI204, EGF, IFI205, NAP1L1, HSPE1, H2-AA, ALOX5AP, PMP22, CD9, BAX, ATP13A2, CKLF, RAN |
| CC | GO:0030670 | Phagocytic vesicle membrane | 11 | 0.00673132 | RAB32, RAB31, ANXA3, H2-Q9, RAB34, H2-Q7, H2-K1, CORO1A, B2M, H2-D1, RAB8B |
| CC | GO:0005794 | Golgi apparatus | 99 | 0.006972647 | EHF, NCF1, PGAP2, H2-K1, CASC4, PEBP1, CTGF, GJA1, FAM198B, RUVBL1, AP1S2, DENND5A, B2M, CAR4, SLC30A7, SCARA3, POSTN, TIPIN, ST6GAL1, COG6, LIG1, SLC35C1, COG3, CYBB, ADAM10, CYBA, WDR77, TPST1, RRAGA, RAB31, SMO, RAB34, MAL, H2-D1, COPB1, RHOBTB3, DPP7, ST3GAL4, DYM, APOE, IGFBP6, CD14, RAB6A, ABCA1, SEC16B, PRNP, B3GALNT1, CAV2, IRGM2, LARP7, CDC6, BST2, EXT1, CALU, ITM2A, PICALM, ITM2C, H2-AB1, ST6GALNAC2, CPQ, AP4E1, OSBPL11, RND3, DSE, PMEPA1, QSOX1, TOPBP1, TMED7, CTSC, SRGN, TMED9, DUSP26, ZDHHC17, 1190002N15RIK, CCNA2, PLSCR1, GAL, MDFIC, KIFC3, MAPRE1, B4GALT6, CRELD2, CRYAB, CD44, SEC23A, SGMS1, ATL1, H2-Q7, HSPD1, AP3M1, NRAS, CHSY1, GCNT2, CD74, SLC35A1, SLC35A4, GOLM1, PLEKHA8, SULF2 |
| CC | GO:0005635 | Nuclear envelope | 22 | 0.006972647 | RRM1, RRM2, UBE2I, CBX3, CAV2, CSE1L, SHISA5, CYBB, TOR1AIP2, LRMP, RTN4, LMNA, ALOX5AP, S100A6, BNIP2, BAX, NSMF, NUP54, APOE, CLIC1, RAN, TMPO |
| CC | GO:0043209 | Myelin sheath | 25 | 0.006972647 | ATP6V1A, WDR1, PEBP1, THY1, RTN4, ACTG1, HSPD1, STIP1, PRDX2, TUBA1B, TUBA1A, PRDX1, CKB, HSPA9, PDIA3, CCT3, HSP90AA1, MDH2, MIF, ATP1B1, TUBB4B, NME1, DLD, CRYAB, SLC25A4 |
| CC | GO:0000790 | Nuclear chromatin | 28 | 0.007239951 | HDAC1, UHRF1, HMGB2, NUCKS1, RBBP4, HIST1H2AE, HIST1H2AD, HIST1H2AG, HIST1H2AC, HIST1H2AB, TIPIN, HIST1H2AO, HIST1H2AN, HIST1H2AI, H2AFY, HIST1H2AH, STAT1, ACTL6A, TCF12, H2AFX, HIST1H2AP, AR, RAD51, NASP, HAT1, RAD17, HNRNPC, EZH2 |
| CC | GO:0005685 | U1 snRNP | 7 | 0.009905362 | SNRPN, SNRPD1, SNRPG, SNRPD3, SNRPF, SNRPC, SNRPB |
| CC | GO:0000775 | Chromosome, centromeric region | 20 | 0.009969148 | AHCTF1, CENPW, CBX3, ZWILCH, DSCC1, MIS12, HJURP, CDCA8, CENPA, ZWINT, NUDCD2, CFDP1, A730008H23RIK, NUF2, BIRC5, KIF2C, BUB3, SPC25, ZFP330, MAD2L1 |
| CC | GO:0031390 | Ctf18 RFC-like complex | 5 | 0.011361159 | RFC5, RFC3, RFC4, RFC2, DSCC1 |
| CC | GO:0042470 | Melanosome | 16 | 0.012592341 | PDIA3, HSP90AA1, ANXA2, TFRC, YWHAB, SERPINF1, RPN1, NAP1L1, CAPG, PDIA4, RAB32, PRDX1, CALU, STOM, RAN, CTSB |
| CC | GO:0005925 | Focal adhesion | 40 | 0.013199291 | RPL3, AHNAK, ITGB5, YWHAB, FBLIM1, CD81, RPL12, ARPC1B, SNAP23, TWF1, THY1, RND3, ACTG1, ICAM1, GJA1, CSRP2, CFL1, ITGAV, B2M, TGM2, HSPA9, PDIA3, PPP1R12A, ANXA1, TPM4, CAV2, ANXA5, ADAM10, CYBA, DAB2, PTPRC, FAP, PALLD, CDH13, SDC1, CD9, VIM, PHLDB2, CD44, DDR2 |
| CC | GO:0005681 | Spliceosomal complex | 19 | 0.015263186 | RBM17, ALYREF, SRSF1, TTF2, HNRNPM, SYNCRIP, LGALS3, DDX39B, SNRPD1, MAGOH, SRSF2, SNRPB2, SNRPD3, HNRNPC, SNRPF, ALYREF2, HNRNPA1, SKIV2L2, SNRPB |
| CC | GO:0000777 | Condensed chromosome kinetochore | 14 | 0.017330407 | AHCTF1, CENPW, ZWILCH, HJURP, ZWINT, NUDCD2, CFDP1, A730008H23RIK, NUF2, BIRC5, KIF2C, BUB3, SPC25, MAD2L1 |
| CC | GO:0071013 | Catalytic step 2 spliceosome | 15 | 0.020125567 | SNRPN, SRSF1, HNRNPM, SYNCRIP, RBMXL1, SNRPD1, MAGOH, SNRPG, SNRPB2, SNRPD3, HNRNPC, SNRPF, HNRNPA1, SKIV2L2, SNRPB |
| CC | GO:0045121 | Membrane raft | 29 | 0.022466051 | RGS19, AHNAK, KCNE3, BCL10, THY1, HSPD1, ICAM1, GJA1, CASP8, EFHD2, STOM, MAL2, CD14, GPM6B, S100A10, ABCA1, PRNP, FCER1G, ANXA2, CAV2, TGFBR1, TGFBR2, BST2, FCGR1, PLSCR1, PTPRC, MAL, CD48, H2-D1 |
| CC | GO:0042612 | MHC class I protein complex | 6 | 0.023775384 | H2-Q9, H2-Q7, H2-K1, H2-L, B2M, H2-D1 |
| CC | GO:0034719 | SMN-Sm protein complex | 6 | 0.023775384 | SNRPD1, SNRPG, GEMIN6, SNRPD3, SNRPF, SNRPB |
| CC | GO:0005663 | DNA replication factor C complex | 4 | 0.024162955 | RFC5, RFC3, RFC4, RFC2 |
| CC | GO:0005813 | Centrosome | 42 | 0.024162955 | PCNA, TUBGCP2, CETN2, VPS4B, SLAIN2, BRCA1, AAAS, WDR43, PKHD1, CDC20, CCNB2, CCNB1, RNF19A, CHEK1, NDN, TOPBP1, SKP1A, CEP55, PLK4, RANBP1, LRRC45, PPP1R12A, SLC16A1, H2AFY, HAUS4, HAUS3, DYNLL1, HAUS1, FBXO31, NME1, RAD51D, TTC8, PSMA1, FAM161A, CDK1, MCM3, TRIM59, KIFC3, MAPRE1, DTL, NFE2L2, ZFP110 |
| CC | GO:0000784 | Nuclear chromosome, telomeric region | 17 | 0.039283335 | PCNA, H2AFY, MCM7, CBX3, RPA1, RPA2, RAD51D, SMCHD1, RAD51, WDR82, HAT1, CDK1, MCM3, MCM4, MCM5, MCM6, MCM2 |
| CC | GO:0070761 | Pre-snoRNP complex | 4 | 0.043640166 | NOP56, NOP58, ZNHIT6, TAF9 |
| CC | GO:0001726 | Ruffle | 14 | 0.043640166 | ANXA2, TESC, FRMD4B, AIF1, AMOT, MTM1, ACAP2, PALLD, RAB34, PDPN, S100A6, INPP5J, S100A11, SNX5 |
| CC | GO:0000794 | Condensed nuclear chromosome | 9 | 0.043819334 | RAD51, NIFK, H2AFX, CHEK1, RPA1, RRS1, TOPBP1, BRCA1, ADD3 |
| CC | GO:0030529 | Intracellular ribonucleoprotein complex | 32 | 0.049335571 | SLBP, SRP19, RPL3, RPL12, MRPL16, BRCA1, MRPL13, SYNCRIP, FBL, XPO1, PABPN1, SNRPD1, RUVBL1, RPL13, SNRPB2, SNRPD3, HNRNPA1, TRIM21, HNRNPA0, NOP56, NOP58, SNRPN, MRPS18B, LARP7, ILF2, HNRNPM, RBMXL1, HNRNPC, SNRPF, SNRPC, LSM14A, SNRPB |
| CC | GO:0005604 | Basement membrane | 14 | 0.049335571 | SPARC, ANXA2, SERPINF1, TRF, FBLN1, PTN, THBS4, LOXL1, CCDC80, CFDP1, TIMP1, TGFBI, FBN1, MATN2 |
| MF | GO:0007049 | Cell cycle | 91 | 4.86388E-12 | CDKN1C, LIN54, AHCTF1, RIF1, MCM7, ZAK, ZWILCH, CETN2, DSCC1, NCAPG2, HJURP, BRCA1, SMC2, CKS1B, CDC20, RGS2, CCND2, CDC23, A730008H23RIK, NUF2, RUVBL1, CHEK1, SPIN1, FBXO5, TRIM21, CEP55, TIPIN, LIG1, H2AFX, HAUS4, HAUS3, HAUS1, KLHL42, CCNA2, TFDP1, PSRC1, RBL1, CINP, CKS2, MCM3, BIRC5, MCM4, GAS1, ERH, MCM5, KIF2C, MCM6, MAPRE1, MCM2, RALB, CDCA3, UHRF1, VPS4B, MIS12, CDCA8, LIN9, KLHL13, ANAPC10, NCAPH, CCNB2, SYCE2, CCNB1, RACGAP1, RBBP4, RBBP8, E2F3, BUB3, ECT2, CDT1, CENPW, UBE2I, UBE2C, USP9X, GADD45A, CDC7, CDC6, ZWINT, FBXO31, ANLN, CDK7, NASP, CDK4, UBE2S, CCNG1, PMP22, CDK1, RAD17, NCAPD3, RAN, SPC25, MAD2L1 |
| MF | GO:0006260 | DNA replication | 33 | 1.36259E-09 | 2810417H13RIK, PCNA, MCM7, PRIM1, DSCC1, BRCA1, RBBP4, POLD2, REV3L, RFC5, CDT1, RFC3, RRM1, RRM2, RFC4, LIG1, RFC2, RMI1, RPA1, RPA2, CDC6, SSRP1, NASP, RPA3, POLE2, CINP, MCM3, MCM4, MCM5, MCM6, SSBP1, DTL, MCM2 |
| MF | GO:0051301 | Cell division | 59 | 2.24293E-08 | AHCTF1, ZWILCH, CETN2, NCAPG2, SMC2, CKS1B, CDC20, CCND2, CDC23, NUF2, RUVBL1, FBXO5, CEP55, TIPIN, LIG1, HAUS4, HAUS3, HAUS1, KLHL42, CCNA2, PSRC1, CINP, CKS2, BIRC5, MCM5, KIF2C, MAPRE1, RALB, CDCA3, VPS4B, MIS12, CDCA8, KLHL13, ANAPC10, NCAPH, CCNB2, SYCE2, CCNB1, RACGAP1, RBBP8, BUB3, ECT2, CENPW, UBE2I, UBE2C, USP9X, CDC7, CDC6, ZWINT, ANLN, CDK7, CDK4, UBE2S, CCNG1, CDK1, NCAPD3, RAN, SPC25, MAD2L1 |
| MF | GO:0007067 | Mitotic nuclear division | 47 | 2.11663E-07 | AHCTF1, CDCA3, ZWILCH, TTYH1, CETN2, MIS12, NCAPG2, CDCA8, KLHL13, ANAPC10, NCAPH, SMC2, CDC20, CCNB2, CCNB1, CDC23, NUF2, RUVBL1, RBBP8, FBXO5, BUB3, CEP55, TIPIN, CENPW, PPP1R12A, UBE2I, UBE2C, USP9X, HAUS4, HAUS3, CDC6, ZWINT, HAUS1, KLHL42, CCNA2, ANLN, PSRC1, CCNG1, SRSF2, CDK1, BIRC5, KIF2C, NCAPD3, MAPRE1, RAN, SPC25, MAD2L1 |
| MF | GO:0006397 | mRNA processing | 46 | 5.30324E-05 | SLBP, RNASEL, DBR1, RNMT, CELF2, CSTF3, SRSF1, CSTF2, TTF2, SYNCRIP, LGALS3, PABPN1, PPP4R2, SNRPD1, PAPOLG, TRA2B, TRA2A, MAGOH, SNRPB2, QK, SNRPD3, ALYREF2, HNRNPA1, SRSF10, APOBEC1, RBM17, PNPT1, ZRANB2, NCBP2, ALYREF, HNRNPM, RBMXL1, DDX39B, PDE12, XRN2, SRSF2, SRSF3, SNRNP27, GEMIN6, CDK12, HNRNPC, SNRPF, TARDBP, SRSF7, SKIV2L2, SNRPB |
| MF | GO:0052697 | Xenobiotic glucuronidation | 8 | 5.30324E-05 | UGT1A10, UGT1A1, UGT1A5, UGT1A2, UGT1A9, UGT1A6A, UGT1A6B, UGT1A7C |
| MF | GO:0051603 | Proteolysis involved in cellular protein catabolic process | 17 | 6.88917E-05 | CTSZ, CTSS, PSMB8, PSMB9, SCPEP1, CTSO, PSMA3, PSMA4, CASP8, PSMA1, FAP, CTSL, CTSK, CTSH, CTSC, LGMN, CTSB |
| MF | GO:0019882 | Antigen processing and presentation | 16 | 0.000172994 | CD74, H2-EB1, H2-K1, H2-L, H2-AA, CTSS, PSMB8, PSMB9, RAB32, CD1D1, FCGRT, RAB34, RAB6A, H2-D1, H2-AB1, RAB8B |
| MF | GO:0070584 | Mitochondrion morphogenesis | 12 | 0.000538089 | SUPV3L1, PID1, PNPT1, MUP19, MUP9, BAX, MUP16, SSBP1, MUP1, MUP12, MUP2, MTM1 |
| MF | GO:0006457 | Protein folding | 24 | 0.0006732 | MESDC2, HSPA9, PDIA3, CCT3, CD74, HSP90AA1, FKBP14, HSPE1, PDIA5, PDRG1, PDIA4, HSPD1, DNAJA1, CCT6A, DNAJB1, TUBA1B, TUBB5, NUDCD2, DNAJB4, FKBP7, QSOX1, PFDN2, CRYAB, PPID |
| MF | GO:0048146 | Positive regulation of fibroblast proliferation | 16 | 0.001625752 | CD74, ANXA2, EGF, WNT5A, FNDC3B, MIF, CDC6, CCNA2, NRAS, CCNB1, CDK4, PDGFD, MYC, PDGFC, GAS6, DDR2 |
| MF | GO:0010888 | Negative regulation of lipid storage | 9 | 0.002027631 | CRP, MUP19, MUP9, MUP16, ITGAV, MUP1, MUP12, MUP2, PTPN2 |
| MF | GO:0006268 | DNA unwinding involved in DNA replication | 7 | 0.00217653 | TOP2A, PURB, RAD51, MCM7, MCM4, MCM6, MCM2 |
| MF | GO:0009813 | Flavonoid biosynthetic process | 9 | 0.003923468 | UGT1A10, UGT1A1, UGT8A, UGT1A5, UGT1A2, UGT1A9, UGT1A6A, UGT1A6B, UGT1A7C |
| MF | GO:0052696 | Flavonoid glucuronidation | 9 | 0.003923468 | UGT1A10, UGT1A1, UGT8A, UGT1A5, UGT1A2, UGT1A9, UGT1A6A, UGT1A6B, UGT1A7C |
| MF | GO:0008380 | RNA splicing | 33 | 0.00412639 | SRSF1, TTF2, SYNCRIP, LGALS3, PPP4R2, SNRPD1, TRA2B, TRA2A, MAGOH, SNRPB2, QK, SNRPD3, ALYREF2, HNRNPA1, SRSF10, RBM17, ZRANB2, NCBP2, ALYREF, HNRNPM, RBMXL1, DDX39B, SRSF2, SRSF3, SNRNP27, GEMIN6, CDK12, HNRNPC, SNRPF, TARDBP, SRSF7, SKIV2L2, SNRPB |
| MF | GO:0002479 | Antigen processing and presentation of exogenous peptide antigen via MHC class I, TAP-dependent | 10 | 0.009253171 | PSMA3, PSMA4, PSMA1, H2-Q9, H2-Q7, H2-K1, B2M, PSMB8, H2-D1, PSMB9 |
| MF | GO:0043032 | Positive regulation of macrophage activation | 8 | 0.009535885 | IL33, CD1D1, IFI204, WNT5A, LGALS9, THBS1, HAMP, HSPD1 |
| MF | GO:0006270 | DNA replication initiation | 9 | 0.009535885 | MCM7, MCM3, MCM4, CDC7, MCM5, MCM6, TOPBP1, CDC6, MCM2 |
| MF | GO:0051028 | mRNA transport | 17 | 0.010547363 | SLBP, AHCTF1, NCBP2, ALYREF, SRSF1, AAAS, NUP160, XPO1, DDX39B, MAGOH, NUP35, SRSF3, QK, NUP54, ALYREF2, HNRNPA1, SRSF7 |
| MF | GO:0010977 | Negative regulation of neuron projection development | 14 | 0.01515374 | H2-K1, RTN4, PTPRG, DAB2, BAG5, DPYSL3, PMP22, INPP5J, VIM, B2M, EPHA3, H2-D1, ITM2C, MAP4K4 |
| MF | GO:0010628 | Positive regulation of gene expression | 43 | 0.040002724 | CRP, PID1, SGMS1, HMGB2, BRCA1, HIF1A, CTGF, GJA1, LAMP3, LMNA, AGR2, CTSH, QK, LGALS9, CCR5, CD34, TRP53INP1, IL33, SEC16B, EGR1, BCL11A, TESC, PTGES3, TCF12, MUP16, SERPINB9, MUP1, MUP12, MUP2, TGFBR1, AR, PLSCR1, MAF, SMO, MUP19, MUP9, LCN2, CDK1, VIM, GAS6, CD44, PF4, NFE2L2 |
| MF | GO:0048661 | Positive regulation of smooth muscle cell proliferation | 15 | 0.040002724 | ABCC4, EGR1, STAT1, CYBA, THBS1, AIF1, HIF1A, TGFBR2, PDGFD, MYC, NAMPT, C3AR1, CDH13, SKP2, TGM2 |
| MF | GO:0042493 | Response to drug | 38 | 0.043402262 | SRP19, MCM7, HMGB2, MGST1, TGFA, PEBP1, PTN, TYMS, ADD3, THBS1, HSPD1, ICAM1, CCNB1, XPO1, CD38, CAR9, B2M, APOBEC1, ABCA1, SS18, HSP90AA1, MMP7, ANXA1, UGT1A1, CHUK, STAT1, MMP2, CYBB, CYBA, TGFBR2, GAL, RAD51, ADORA2A, LOX, CDK4, LCN2, CDK1, PLIN2 |

Table S5. The results of KEGG analysis of the CP-associated DEGs.

| ID | Term | Count | adj.P | Genes |
| --- | --- | --- | --- | --- |
| mmu03030 | DNA replication | 20 | 3.13439E-11 | RFC5, RFC3, RFC4, PCNA, RNASEH2A, MCM7, LIG1, RFC2, PRIM1, RPA1, RPA2, RPA3, POLE2, POLD2, MCM3, MCM4, MCM5, MCM6, SSBP1, MCM2 |
| mmu04110 | Cell cycle | 32 | 3.10939E-08 | CDKN1C, PCNA, MCM7, YWHAB, HDAC1, ANAPC10, CDC20, CCNB2, CCNB1, CDC23, CCND2, MYC, CHEK1, E2F3, BUB3, SKP2, SKP1A, GADD45A, CDC7, CDC6, CCNA2, CDK7, RBL1, TFDP1, CDK4, CDK1, MCM3, MCM4, MCM5, MCM6, MCM2, MAD2L1 |
| mmu03430 | Mismatch repair | 11 | 6.5814E-05 | RFC5, RFC3, RFC4, PCNA, LIG1, RFC2, RPA3, POLD2, RPA1, RPA2, SSBP1 |
| mmu03040 | Spliceosome | 27 | 8.52583E-05 | TCERG1, SRSF1, SNRPD1, TRA2B, TRA2A, MAGOH, SNRPB2, SNRPD3, ALYREF2, HNRNPA1, SRSF10, RBM17, NCBP2, ALYREF, HNRNPM, GM5803, RBMXL1, DDX39B, SRSF2, SNRPG, SRSF3, SNRNP27, HNRNPC, SNRPF, SNRPC, SRSF7, SNRPB |
| mmu04145 | Phagosome | 31 | 0.000116233 | COLEC12, ATP6V1A, TFRC, ITGB5, NCF1, H2-M3, H2-Q7, H2-K1, THBS1, CORO1A, THBS4, CTSS, ACTG1, TUBA1B, TUBB5, TUBA1A, CLEC7A, CTSL, MRC1, ITGAV, CD14, H2-EB1, SFTPD, CYBA, TUBB4B, H2-AA, FCGR1, FCGR3, TUBB2A, H2-D1, H2-AB1 |
| mmu03420 | Nucleotide excision repair | 14 | 0.00024618 | RFC5, RFC3, RFC4, PCNA, LIG1, RFC2, CETN2, RPA1, RPA2, GTF2H2, CDK7, RPA3, POLE2, POLD2 |
| mmu00040 | Pentose and glucuronate interconversions | 11 | 0.001989212 | UGT1A10, UGDH, UGT1A1, UGT1A5, AKR1A1, UGT1A2, UGT1A9, UGT1A6A, UGT1A6B, UGT1A7C, AKR1B8 |
| mmu05150 | Staphylococcus aureus infection | 13 | 0.004022185 | C1QB, C1QA, H2-EB1, CFH, CFI, H2-AA, ICAM1, C4B, FCGR1, FCGR3, C3AR1, C1QC, H2-AB1 |
| mmu04612 | Antigen processing and presentation | 17 | 0.004148473 | PDIA3, CD74, H2-EB1, HSP90AA1, NFYA, H2-Q7, H2-M3, H2-K1, H2-AA, CTSS, CTSL, PSME1, B2M, H2-D1, LGMN, CTSB, H2-AB1 |
| mmu05166 | HTLV-I infection | 37 | 0.004857822 | ATF2, PCNA, H2-M3, H2-Q7, H2-K1, ADCY7, ANAPC10, ICAM1, CDC20, NRAS, CDC23, XPO1, CCND2, MYC, MYB, POLD2, CHEK1, E2F3, BUB3, TRP53INP1, EGR1, RANBP1, H2-EB1, CHUK, IL1R1, WNT5A, H2-AA, TGFBR1, TGFBR2, CDK4, POLE2, BAX, SLC25A4, H2-D1, RAN, MAD2L1, H2-AB1 |
| mmu05322 | Systemic lupus erythematosus | 24 | 0.004857822 | C1QB, C1QA, HIST1H2AO, H2-EB1, HIST1H2AN, H2AFY, HIST1H2AI, HIST1H2AH, H2AFX, HIST1H2AP, H2-AA, C4B, FCGR1, SNRPD1, HIST1H2AE, HIST1H2AD, HIST1H2AG, SNRPD3, TRIM21, HIST1H2AC, HIST1H2AB, C1QC, H2-AB1, SNRPB |
| mmu00053 | Ascorbate and aldarate metabolism | 9 | 0.00769509 | UGT1A10, UGDH, UGT1A1, UGT1A5, UGT1A2, UGT1A9, UGT1A6A, UGT1A6B, UGT1A7C |
| mmu03410 | Base excision repair | 10 | 0.009901945 | PARP3, PCNA, LIG1, PARP2, TDG, POLE2, POLD2, GM21596, HMGB1, UNG |
| mmu04115 | p53 signaling pathway | 14 | 0.01091071 | RRM2, APAF1, GADD45A, SHISA5, THBS1, CCNB2, CCNB1, CASP8, CCND2, CDK4, CCNG1, CHEK1, CDK1, BAX |
| mmu00983 | Drug metabolism - other enzymes | 12 | 0.01091071 | UGT1A10, UGT1A1, CES1E, CES2G, IMPDH2, UGT1A5, HPRT, UGT1A2, UGT1A9, UGT1A6A, UGT1A6B, UGT1A7C |

Table S6. The results of GO analysis of the KRT8-correlated DEGs.

| Category | ID | Term | Count | adj.P | Genes |
| --- | --- | --- | --- | --- | --- |
| BP | GO:0098609 | Cell-cell adhesion | 14 | 0.01276798 | LAD1, YWHAZ, LASP1, CEACAM1, KRT18, GPRC5A, EPCAM, FLRT3, PERP, S100P, TAGLN2, SFN, S100A11, EPHA2 |
| BP | GO:0007586 | Digestion | 7 | 0.04161011 | CAPN8, AKR1B10, NMU, TFF3, TFF2, TFF1, CTSE |
| CC | GO:0070062 | Extracellular exosome | 74 | 1.5355E-10 | LGALS3BP, LAD1, SLC44A4, PSCA, MTMR11, SLC2A1, WFDC2, LGALS3, LASP1, CAPN5, CHMP1A, AOX1, MUC13, CTSE, CLIC1, BCAS1, PLS1, GPX2, PARP4, ITGA3, ANXA13, SLC6A14, ACTN4, TMC5, YWHAZ, SERPINB5, MUC5AC, ANO1, CEACAM1, C1ORF116, GPRC5A, SLPI, CEACAM5, TFF3, TFF2, DSG2, TAGLN2, EZR, CLRN3, SDC4, SLC1A1, FGL2, FUT3, RAB25, TSPAN8, S100A16, EPCAM, MAL2, GCNT3, SFN, S100A14, MUC4, TSPAN1, S100A11, DUOX2, SLC12A2, CYP2J2, NQO1, MARCKSL1, JUP, GSS, RAB27B, PDZK1IP1, TSPAN15, KRT19, PTPRC, KRT18, AKR1B10, EIF6, FXYD3, LCN2, SDC1, S100P, SERPINI1 |
| CC | GO:0016324 | Apical plasma membrane | 15 | 0.00056869 | SLC12A2, SLC1A1, ANXA13, SLC2A1, RAB27B, SLC6A20, ANO1, CEACAM1, EPCAM, MAL2, MUC13, DSG2, EZR, KCNK1, DUOX2 |
| CC | GO:0005925 | Focal adhesion | 17 | 0.00063343 | ZNF185, JUP, SDC4, ITGA3, ITGA2, ACTN4, ARHGAP26, YWHAZ, EFNB2, FERMT1, LASP1, PTPRC, FLRT3, CAPN5, SDC1, EZR, EPHA2 |
| CC | GO:0005615 | Extracellular space | 34 | 0.00073903 | LGALS3BP, PTPRR, LAMC2, MSLN, KLK7, WFDC2, KLK6, LGALS4, LGALS3, FLRT3, LIPH, AGR2, MUC13, SFN, APOL1, CLIC1, MUC4, S100A11, SRGN, ANXA13, ACTN4, YWHAZ, SERPINB5, MUC5AC, BMP4, SLPI, CEACAM6, TCN1, LCN2, TFF3, TFF2, TFF1, SERPINI1, EZR |
| CC | GO:0005913 | Cell-cell adherens junction | 15 | 0.00073903 | LAD1, JUP, YWHAZ, LASP1, KRT18, CDH3, GPRC5A, S100P, TAGLN2, SFN, EZR, CLIC1, S100A11, CD200, EPHA2 |
| CC | GO:0005887 | Integral component of plasma membrane | 33 | 0.00383986 | SDC4, SLC1A1, SLC2A1, MST1R, TNFRSF11A, SLC6A20, EFNA4, EFNB2, TSPAN8, FLRT3, VSIG2, EPCAM, PERP, MUC4, TSPAN1, SLC12A2, SLC6A14, TM4SF5, TM4SF1, TSPAN15, CEACAM1, PTPRC, GPRC5A, CEACAM6, FXYD3, CEACAM5, SDC1, KCNK1, MET, TNFRSF21, CD200, LY6E, EPHA2 |
| CC | GO:0031982 | Vesicle | 8 | 0.01771329 | GPRC5A, SLC6A14, TAGLN2, EZR, KCNN4, TSPAN1, CLIC1, MUC4 |
| MF | GO:0098641 | Cadherin binding involved in cell-cell adhesion | 14 | 0.00800192 | LAD1, JUP, YWHAZ, LASP1, KRT18, GPRC5A, EPCAM, S100P, TAGLN2, SFN, EZR, CLIC1, S100A11, EPHA2 |
