# Supplementary material for: Keratin 8 Is an Inflammation-Induced and Prognosis-Related Marker for Pancreatic Adenocarcinoma
Source: Dis Markers. 2022 Jul 27;2022:8159537. doi: 10.1155/2022/8159537 (PMC9359862; doi:10.1155/2022/8159537)
Supplement: Supplementary Materials — Additional file 1: Figure S1. Figure 1: illustration of the work flow diagram. Additional file 2: Figure S2: presentation of the DEGs of murine AP models. The result was visualized by hierarchical cluster heatmaps. (A) GSE3644. (B) GSE109227. (C) GSE121038. The gradual change from red to blue represents the changes of gene expression from high to low. The white color represents no difference in gene expression. (D) Venn diagrams for the intersection of DEGs among different datasets. (E) GO analysis for the DEGs in the intersection. (F) KEGG analysis for the DEGs in the intersection. Additional file 3: Figure S3: presentation of the DEGs of murine CP models. The result was visualized by hierarchical cluster heatmaps. (A) Jackson mice group in GSE41418. (B) Harlan mice group in GSE41418. (C) Venn diagrams for the intersection of DEGs between different datasets. (D) GO analysis for the DEGs in the intersection. (E) KEGG analysis for the DEGs in the intersection. Additional file 4: Figure S4: presentation of the expression of some genes that were not influenced by pancreatitis in GSE40895. Additional file 5: Figure S5: the interaction between KRT8 and other genes in correlation analysis. Additional file 6: Figure S6: illustration of the genes involved in different pathways retrieved in PathCards database. The gradual change from red to blue represents the changes of relevance score from high to low. The white colour represents the genes that have no significant connection with the pathways. Additional file 7: Figure S7: presentation of the genes with the top three relevance scores from high to low in the pathways involved in inflammation process provided by PathCards database. (A) Innate immune system. (B) Interferon. (C) Interleukin. (D) B cell receptor signaling pathway. (E) NF-κB signaling pathway. ∗∗∗P < 0.001. ∗∗P < 0.01. Additional file 8: Figure S8: presentation of the genes with the top three relevance scores from high to low in the pathways involved in cell via [file 8159537.f1.zip › Table S8.docx]

Table S8. Demographic details for the patients involved in the study.

Patients involved in Western blot and qPCR assays.

| Patients | Gender | Age | Pathological Diagnosis | N classification | T classification | M classification |
| --- | --- | --- | --- | --- | --- | --- |
| 1* | Male | 58 | Poorly differentiated pancreatic ductal adenocarcinoma | N1 | T2 | M0 |
| 2* | Female | 60 | Moderately differentiated pancreatic ductal adenocarcinoma | N1 | T3 | M0 |
| 3* | Male | 49 | Highly differentiated pancreatic ductal adenocarcinoma | N1 | T3 | M0 |
| 4* | Female | 41 | Moderately differentiated pancreatic ductal adenocarcinoma | N1 | T3 | M0 |
| 5* | Female | 57 | Highly differentiated pancreatic ductal adenocarcinoma | N1 | T2 | M0 |
| 6* | Male | 61 | Moderately differentiated pancreatic ductal adenocarcinoma | N1 | T2 | M0 |
| 7* | Male | 64 | Moderately differentiated pancreatic ductal adenocarcinoma | N1 | T3 | M0 |
| 8* | Female | 73 | Moderately differentiated pancreatic ductal adenocarcinoma | N1 | T3 | M0 |
| 9* | Female | 49 | Poorly differentiated pancreatic ductal adenocarcinoma | N1 | T3 | M0 |
| 10* | Female | 59 | Moderately differentiated pancreatic ductal adenocarcinoma | N1 | T3 | M0 |

Patients involved in IHC analysis.

| Patients | Gender | Age | Pathological Diagnosis | N classification | T classification | M classification |
| --- | --- | --- | --- | --- | --- | --- |
| 1 | Female | 63 | Moderately differentiated pancreatic ductal adenocarcinoma | N1 | T2 | M0 |
| 2 | Female | 59 | Moderately differentiated pancreatic ductal adenocarcinoma | N1 | T2 | M0 |
| 3 | Male | 55 | Moderately differentiated pancreatic ductal adenocarcinoma | N1 | T2 | M0 |
| 4 | Male | 58 | Moderately differentiated pancreatic ductal adenocarcinoma | N1 | T2 | M0 |
| 5 | Female | 44 | Poorly differentiated pancreatic ductal adenocarcinoma | N1 | T3 | M0 |
| 6 | Male | 59 | Moderately differentiated pancreatic ductal adenocarcinoma | N1 | T2 | M0 |
| 7 | Male | 54 | Moderately differentiated pancreatic ductal adenocarcinoma | N1 | T3 | M0 |
| 8 | Male | 55 | Highly differentiated pancreatic ductal adenocarcinoma | N1 | T2 | M0 |
| 9 | Male | 87 | Poorly differentiated pancreatic ductal adenocarcinoma | N1 | T3 | M0 |
| 10 | Male | 46 | Highly differentiated pancreatic ductal adenocarcinoma | N1 | T2 | M0 |
| 11 | Female | 43 | Moderately differentiated pancreatic ductal adenocarcinoma | N1 | T2 | M0 |
| 12 | Male | 55 | Highly differentiated pancreatic ductal adenocarcinoma | N1 | T2 | M0 |
| 13 | Female | 49 | Poorly differentiated pancreatic ductal adenocarcinoma | N1 | T3 | M0 |
| 14 | Female | 59 | Poorly differentiated pancreatic ductal adenocarcinoma | N1 | T3 | M0 |
| 15 | Male | 57 | Highly differentiated pancreatic ductal adenocarcinoma | N1 | T3 | M0 |
| 16 | Male | 66 | Highly differentiated pancreatic ductal adenocarcinoma | N1 | T2 | M0 |
| 17 | Female | 51 | Moderately differentiated pancreatic ductal adenocarcinoma | N1 | T3 | M0 |
| 18 | Male | 66 | Poorly differentiated pancreatic ductal adenocarcinoma | N1 | T2 | M0 |
| 19 | Female | 45 | Moderately differentiated pancreatic ductal adenocarcinoma | N1 | T2 | M0 |
| 20 | Male | 45 | Moderately differentiated pancreatic ductal adenocarcinoma | N1 | T3 | M0 |
| 21 | Male | 69 | Poorly differentiated pancreatic ductal adenocarcinoma | N1 | T3 | M0 |
| 22 | Female | 68 | Moderately differentiated pancreatic ductal adenocarcinoma | N1 | T3 | M0 |
| 23 | Female | 75 | Highly differentiated pancreatic ductal adenocarcinoma | N1 | T2 | M0 |
| 24 | Female | 29 | Negative exploration | - | - | - |
| 25 | Male | 55 | Negative exploration | - | - | - |
| 26 | Female | 47 | Negative exploration | - | - | - |
| 27 | Female | 41 | Negative exploration | - | - | - |
| 28 | Male | 39 | Negative exploration | - | - | - |
| 29 | Female | 63 | Negative exploration | - | - | - |
| 30 | Female | 33 | Negative exploration | - | - | - |
| 31 | Male | 41 | Negative exploration | - | - | - |
| 32 | Male | 40 | Negative exploration | - | - | - |
| 33 | Female | 25 | Negative exploration | - | - | - |
| 34 | Female | 69 | Negative exploration | - | - | - |
| 35 | Female | 35 | Negative exploration | - | - | - |
| 36 | Male | 66 | Chronic pancreatitis | - | - | - |
| 37 | Female | 59 | Chronic pancreatitis | - | - | - |
| 38 | Male | 61 | Chronic pancreatitis | - | - | - |
| 39 | Female | 61 | Chronic pancreatitis | - | - | - |
| 40 | Male | 79 | Chronic pancreatitis | - | - | - |
| 41 | Male | 84 | Chronic pancreatitis | - | - | - |
| 42 | Male | 39 | Chronic pancreatitis | - | - | - |
| 43 | Female | 56 | Chronic pancreatitis | - | - | - |
| 44 | Female | 70 | Chronic pancreatitis | - | - | - |
| 45 | Male | 56 | Chronic pancreatitis | - | - | - |
| 46 | Male | 52 | Chronic pancreatitis | - | - | - |
| 47 | Male | 59 | Chronic pancreatitis | - | - | - |
| 48 | Male | 72 | Chronic pancreatitis | - | - | - |
| 49 | Male | 63 | Chronic pancreatitis | - | - | - |
| 50 | Female | 55 | Chronic pancreatitis | - | - | - |
| 51 | Male | 59 | Chronic pancreatitis | - | - | - |
| 52 | Female | 50 | Chronic pancreatitis | - | - | - |
| 53 | Male | 45 | Chronic pancreatitis | - | - | - |
| 54 | Male | 60 | Chronic pancreatitis | - | - | - |
| 55 | Male | 52 | Chronic pancreatitis | - | - | - |
| 56 | Female | 72 | Chronic pancreatitis | - | - | - |
| 57 | Male | 74 | Chronic pancreatitis | - | - | - |
| 58 | Male | 55 | Chronic pancreatitis | - | - | - |
| 59 | Male | 79 | Chronic pancreatitis | - | - | - |
| 60 | Female | 74 | Chronic pancreatitis | - | - | - |
| 61 | Male | 44 | Chronic pancreatitis | - | - | - |
| 62 | Female | 53 | Chronic pancreatitis | - | - | - |

* Normal tissue and cancerous tissue were both used for analysis.

- Not applicable.
